# Supplementary material for: A MAPK/miR-29 Axis Suppresses Melanoma by Targeting MAFG and MYBL2
Source: Cancers (Basel). 2021 Mar 19;13(6):1408. doi: 10.3390/cancers13061408 (PMC8003541; doi:10.3390/cancers13061408)
Supplement: Supplementary file 1 [file cancers-13-01408-s001.zip › File S1 - Uncropped Western Blots.pdf]

Uncropped Western Blots

Figure 1B HSP90

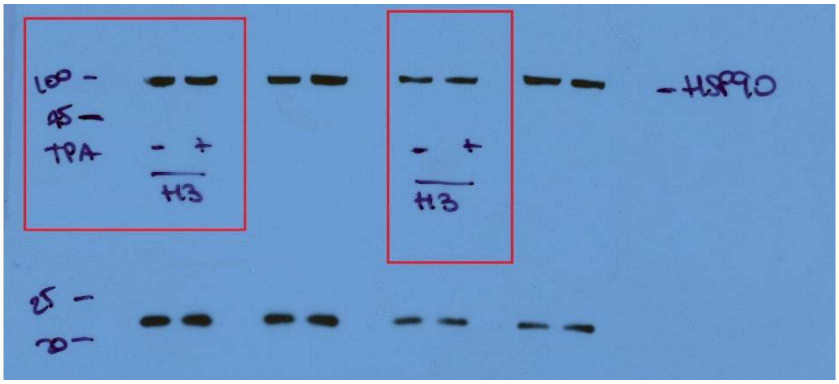

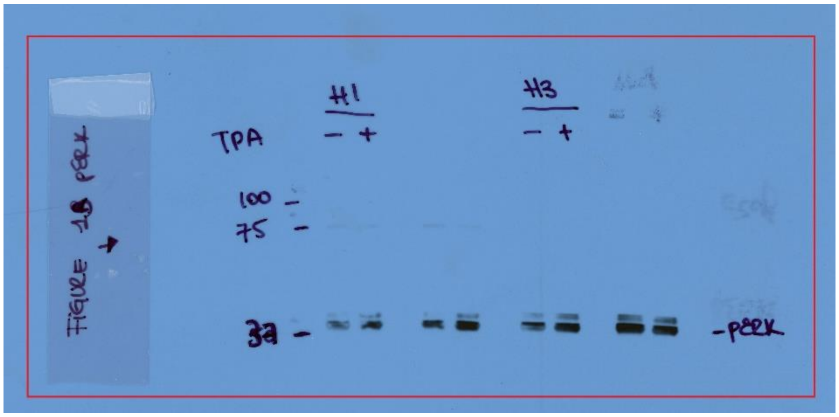

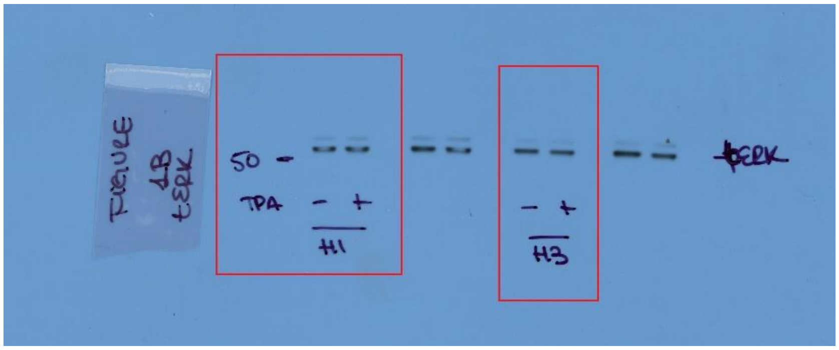

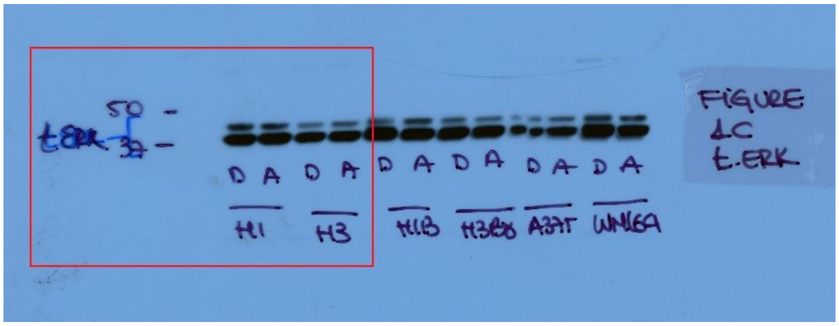
50
tERK
37
D A D A D A D A D A D A
H1 H3 H1B H3B A375 WM164
FIGURE 1C
t.ERK

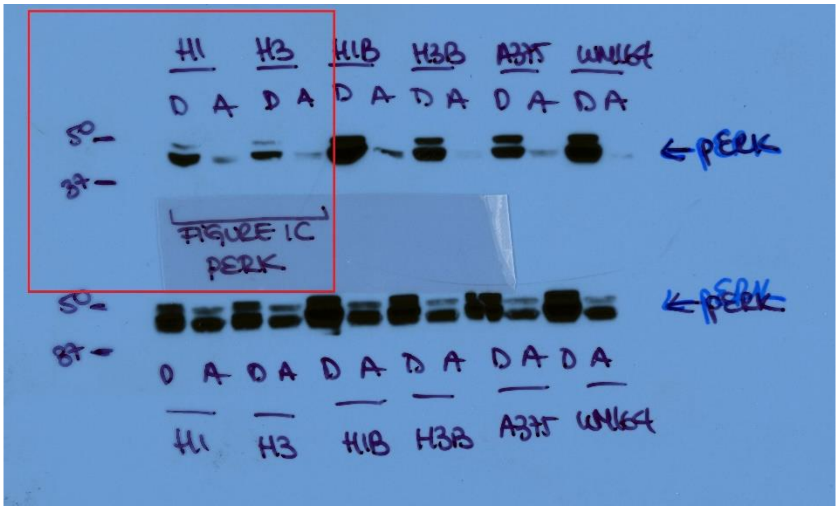
H1 H3 H1B H3B A375 WM164
D A D A D A D A D A D A
50
37
pERK
FIGURE 1C
PERK
50
37
tERK
D A D A D A D A D A D A
H1 H3 H1B H3B A375 WM164

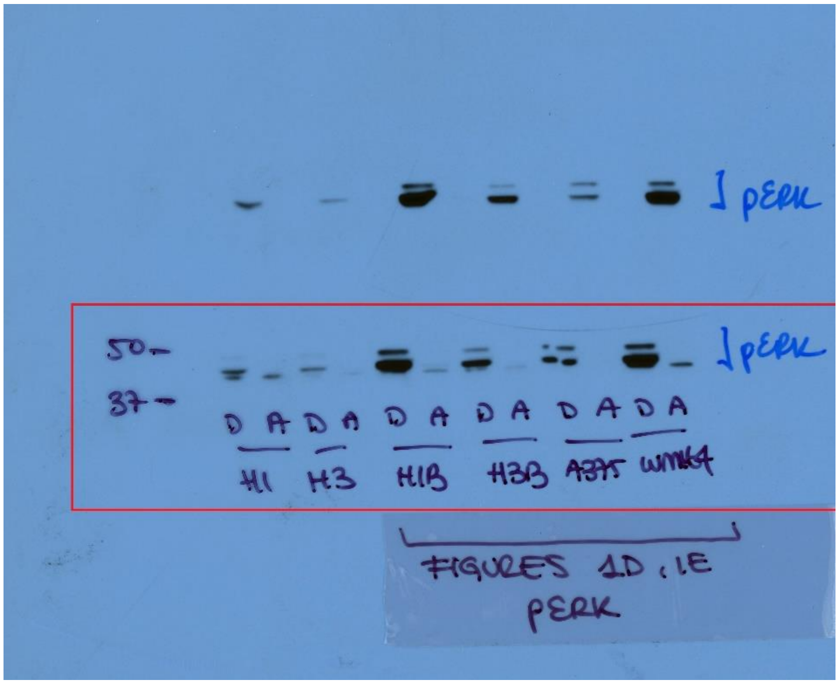
pERK
50
37
pERK
D A D A D A D A D A D A
H1 H3 H1B H3B A375 WM164
FIGURES 1D, 1E
PERK

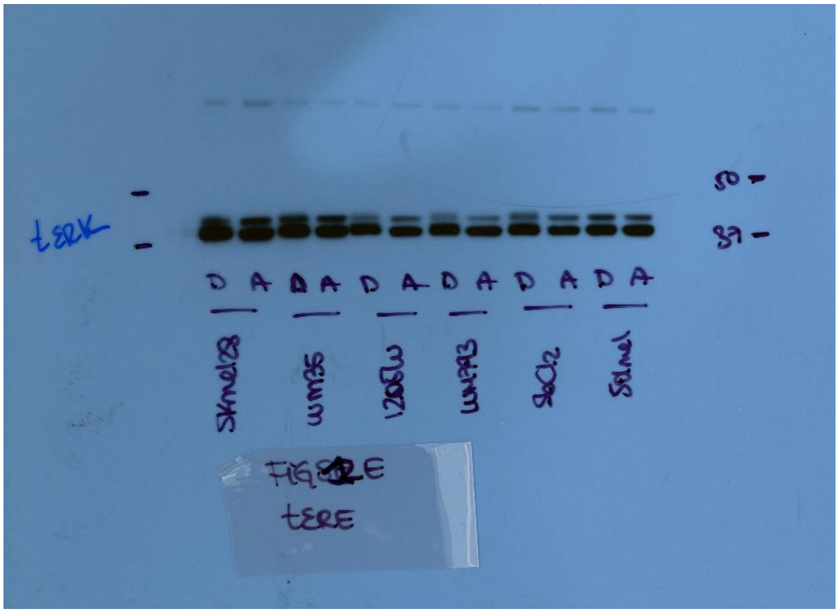

tERK
50
37
SKmel28
WM35
1205Lu
WM793
Sbcl2
501mel
FIGURE tERK

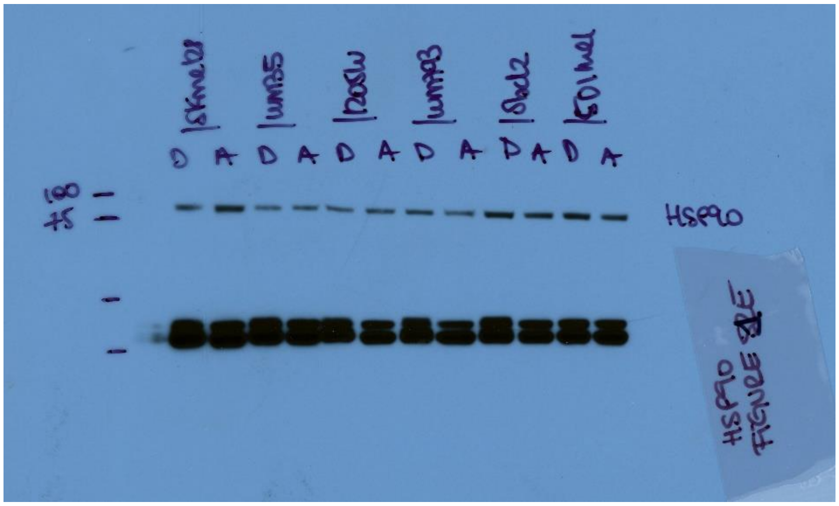

SKmel28
WM35
1205Lu
WM793
Sbcl2
501mel
HSP90
HSP90 FIGURE

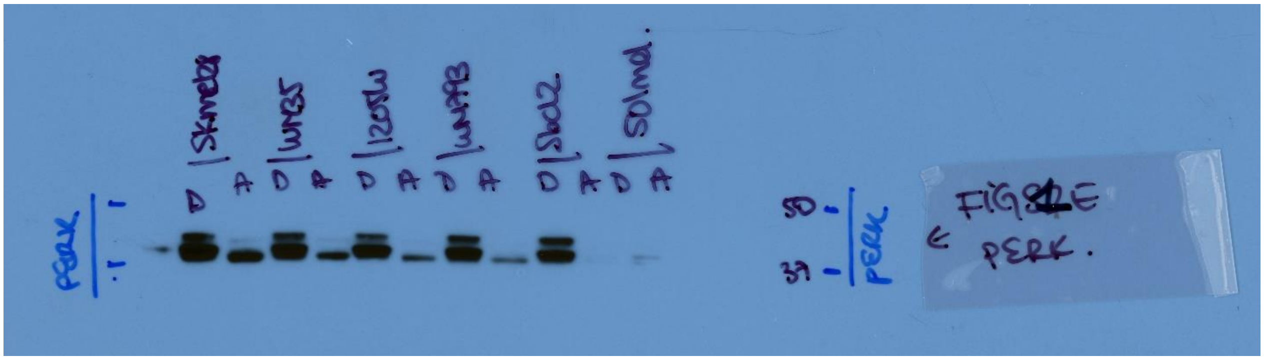

pERK
SKmel28
WM35
1205Lu
WM793
Sbcl2
501mel.
50
37
FIGURE pERK.

Figure 5A HSP90

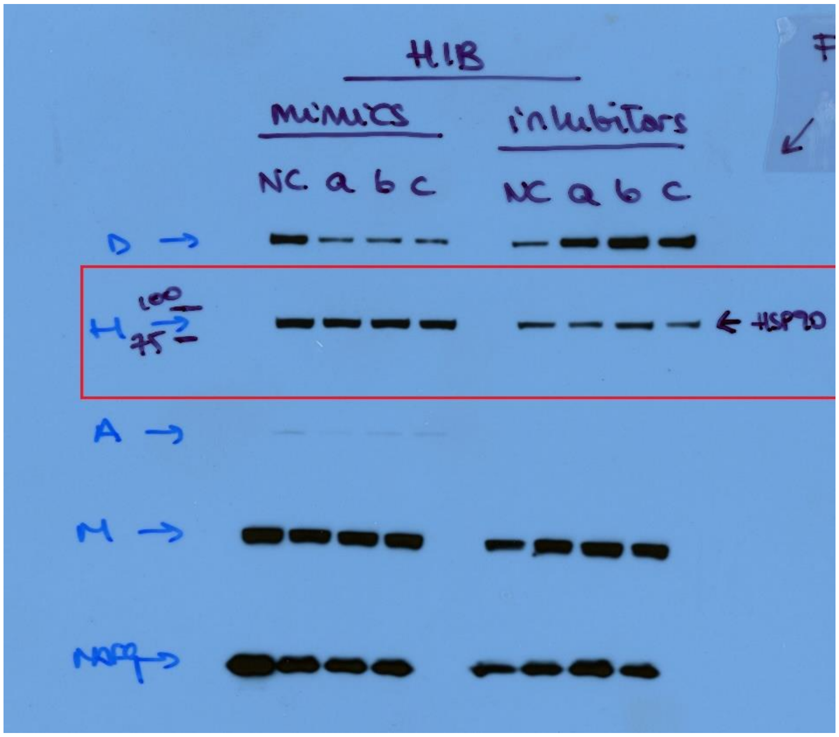

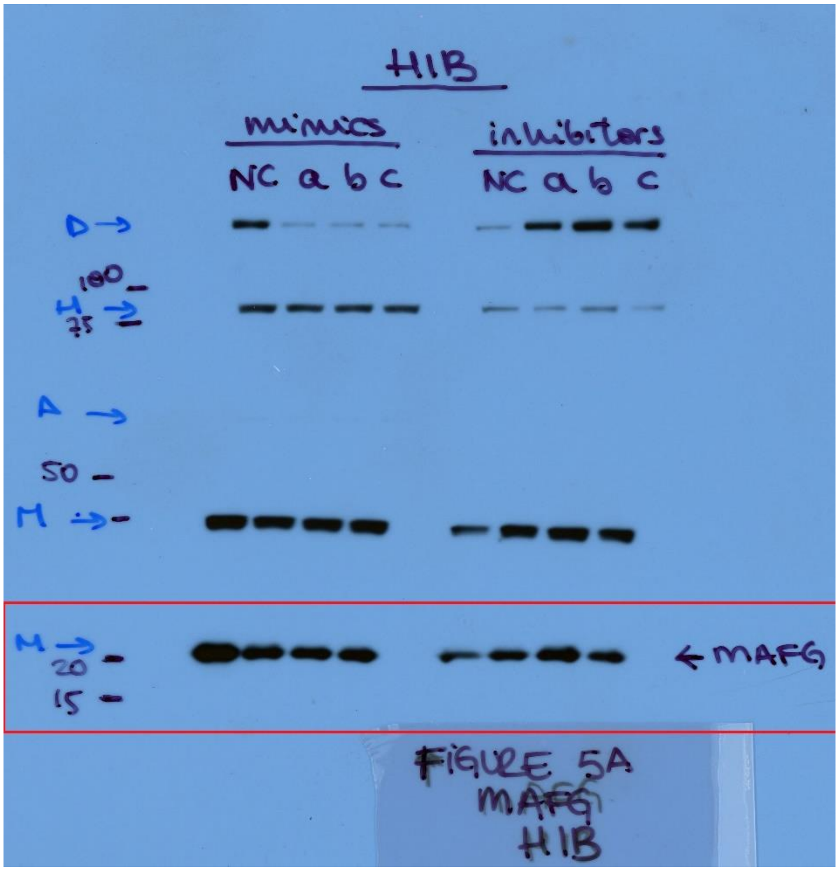

Figure 5B HSP90

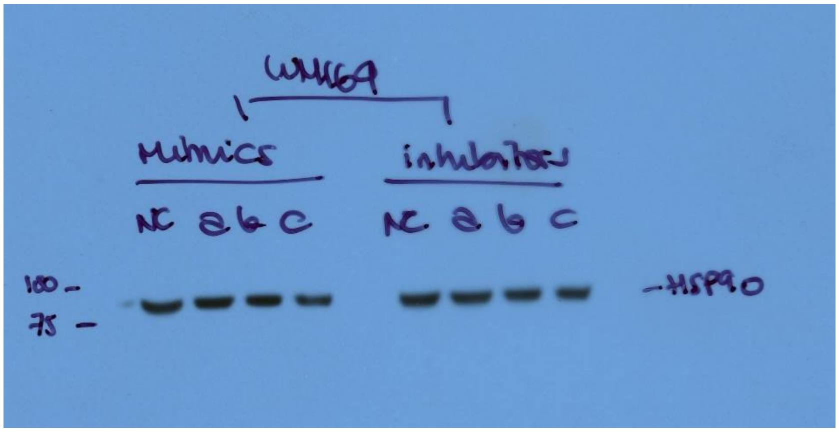

Figure 5B MAFG

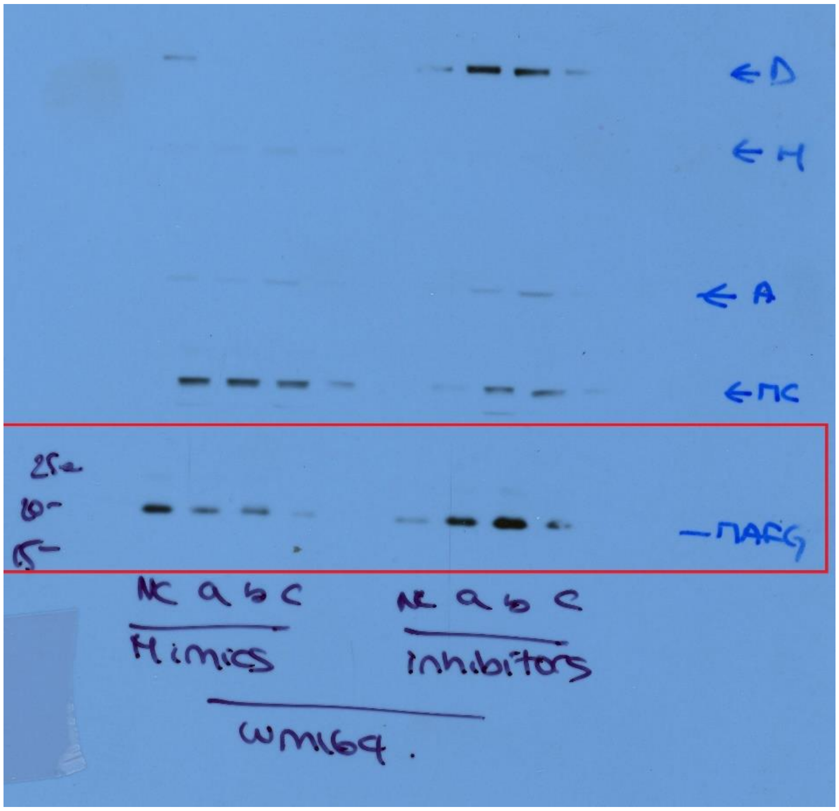

Figure 5E

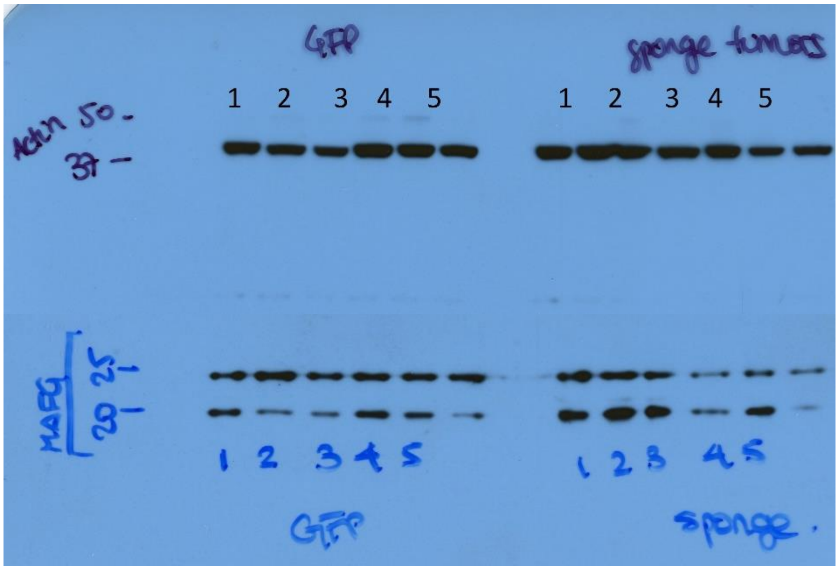

Figure 5G

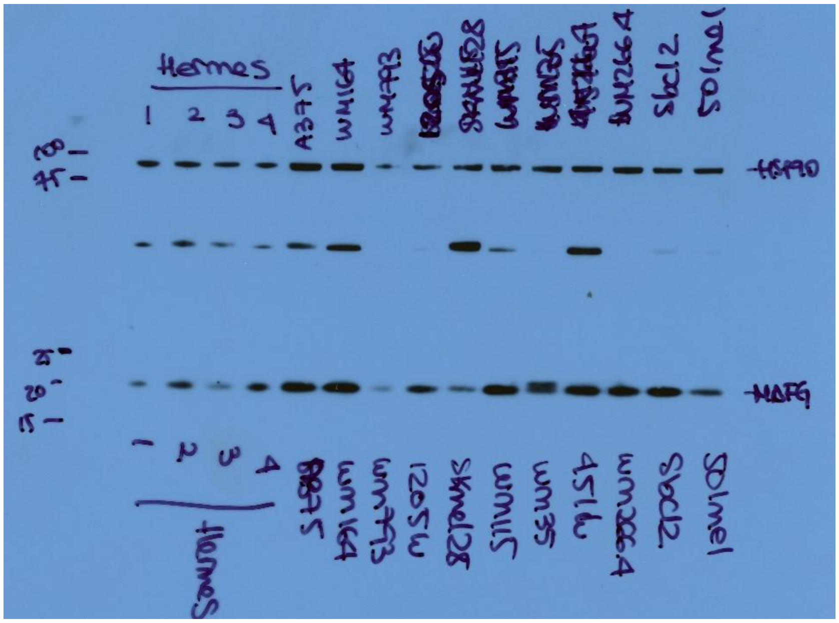

Figure 5K

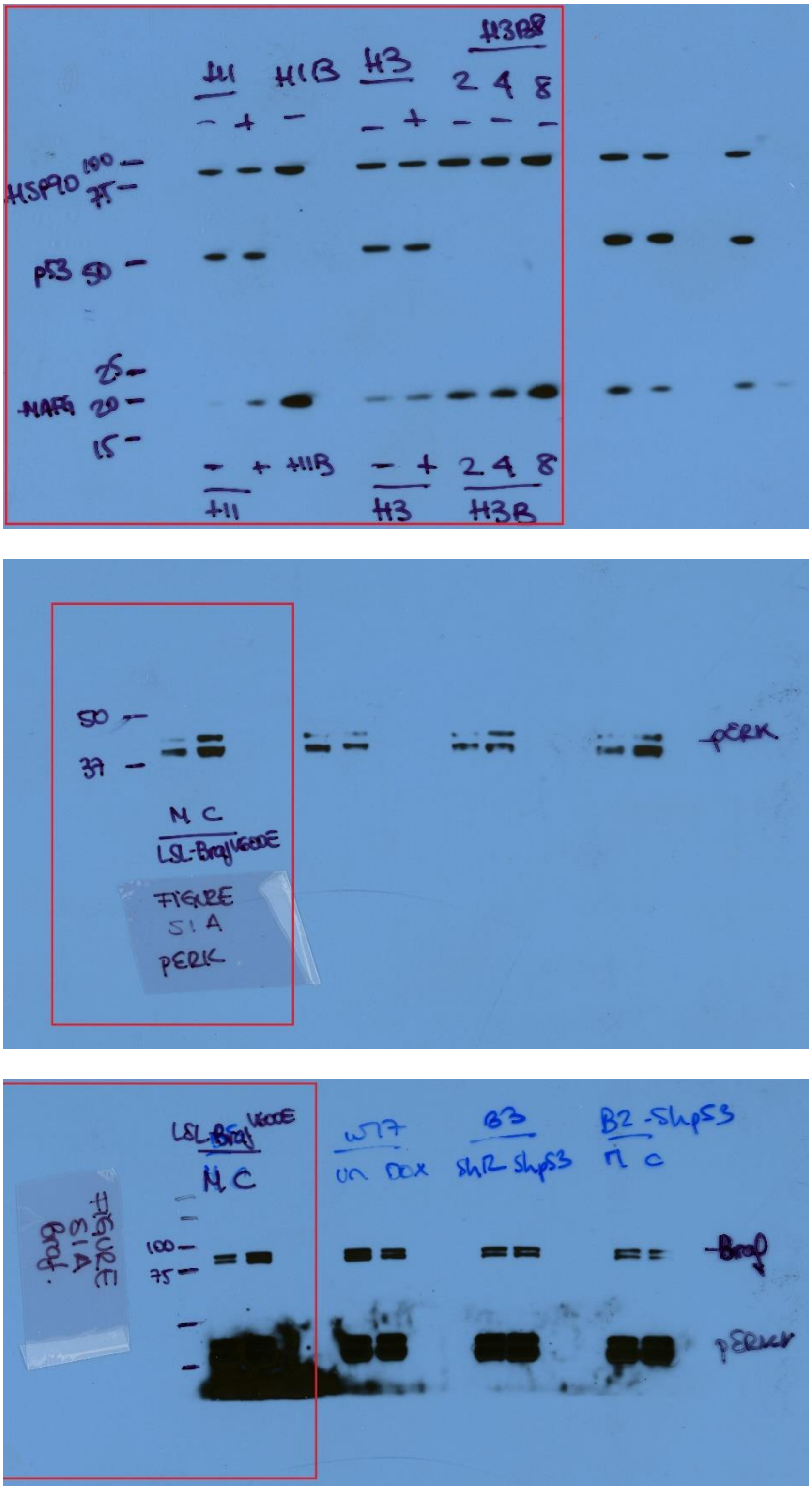

HSP90
p53
MAFG
pERK
FIGURE S1A pERK
LSL-Braf V600E
M C
W77
un DOX
B3
shR shp53
B2-shp53
Braf
FIGURE S1A Braf

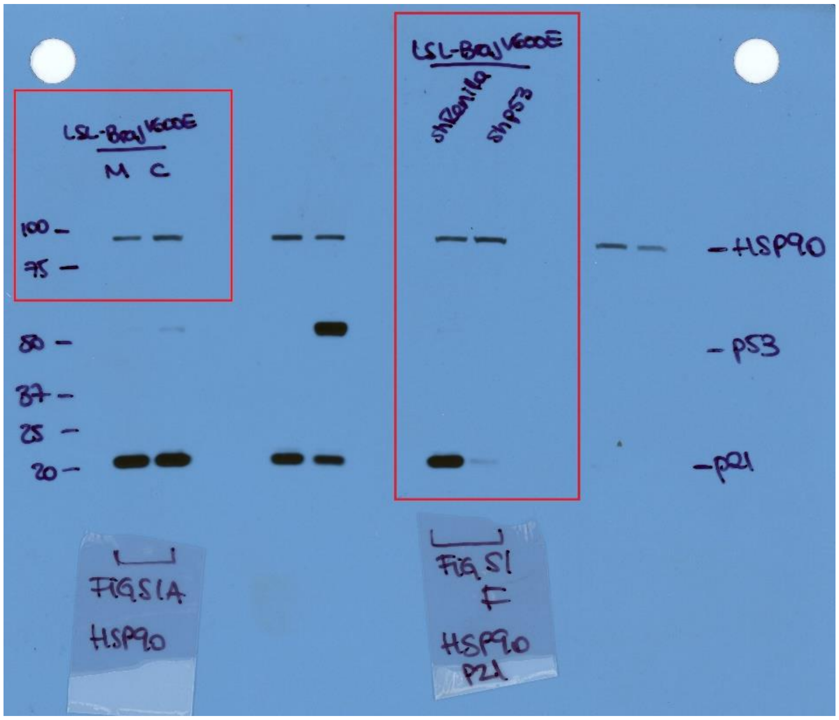
LSL-Braf V600E
M C
100
75
37
25
20
shRenilla
shp53
HSP90
p53
p21
FIG S1A
HSP90
FIG S1
F
HSP90
P21

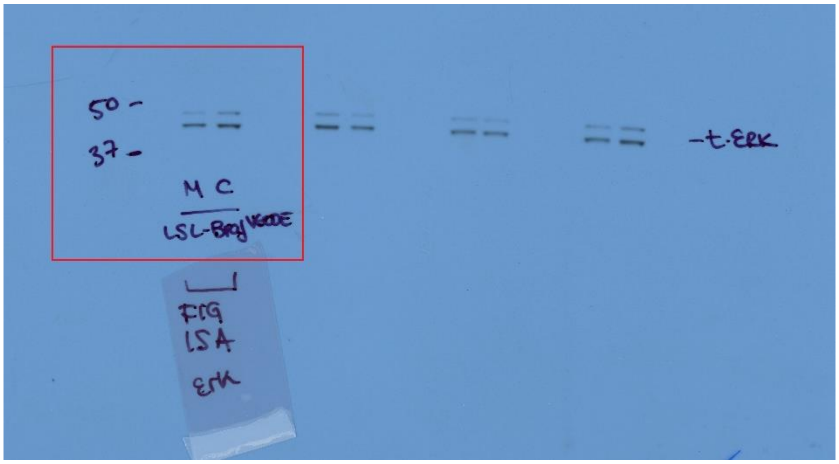
50
37
t-ERK
M C
LSL-Braf V600E
FIG
Erk

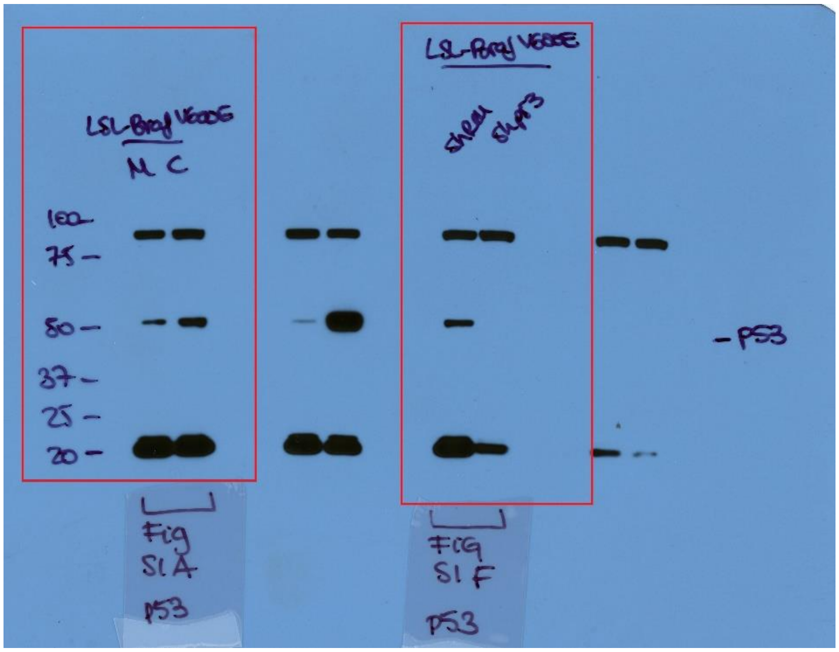
LSL-Braf V600E
N C
100
75
50
37
25
20
LSL-Braf V600E
p53
Fig S1 A p53
Fig S1 F p53

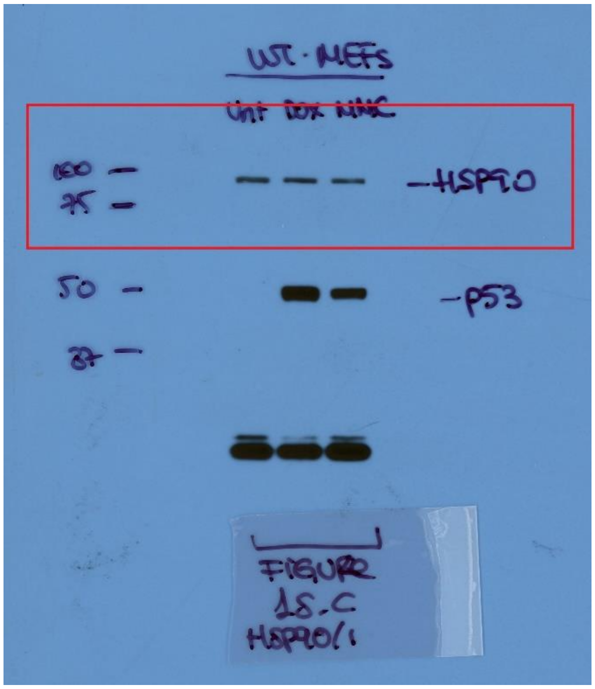
WT·MEFS
Unt Dox NAC
100
75
HSP90
50
p53
37
FIGURE

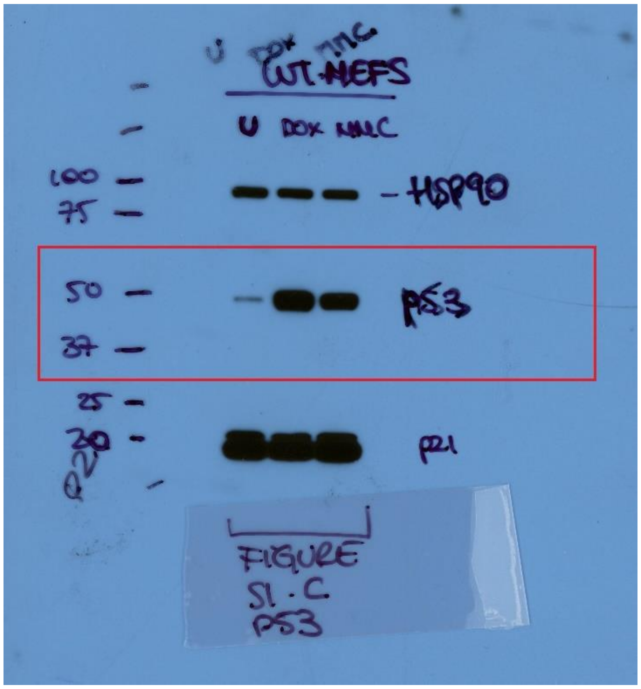
WT·MEFS
U Dox NAC
100
75
HSP90
50
37
p53
25
20
p21
FIGURE S1.C p53

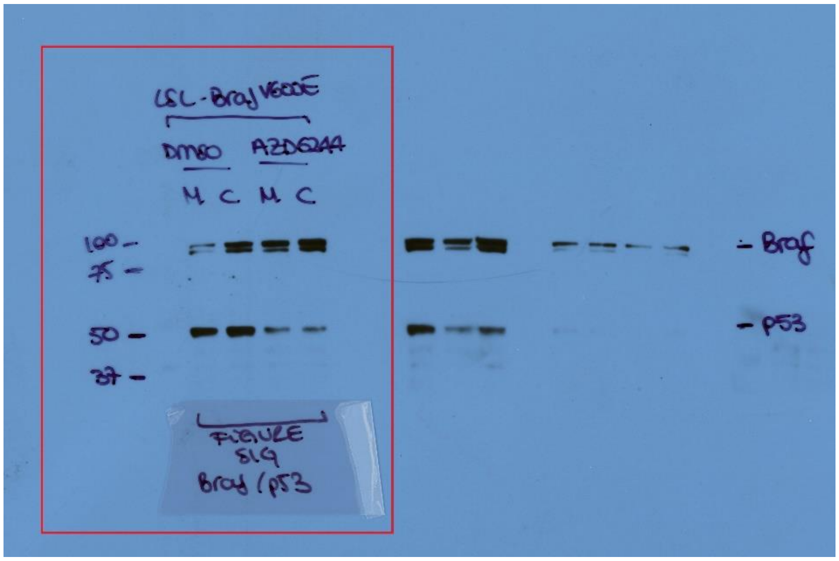
LSL-Braf V600E
DMSO
AZD6244
M C M C
100
75
50
37
Braf
p53
FIGURE
S19
Braf / p53

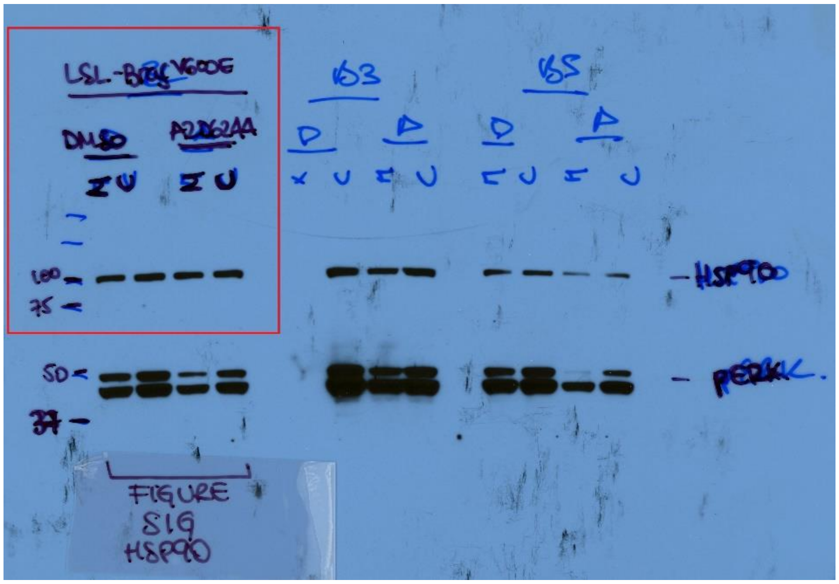
LSL-Braf V600E
DMSO
AZD6244
163
165
D
A
100
75
50
37
HSP90
pERK
FIGURE
S19
HSP90

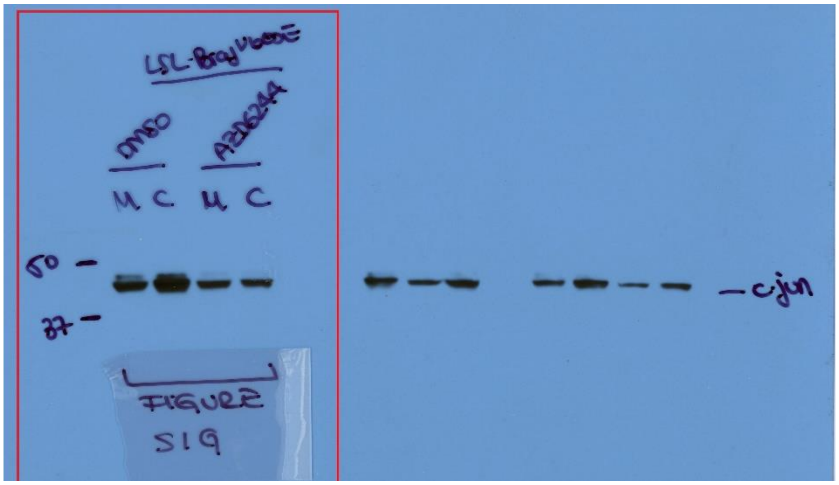
LSL-Braf V600E
DMSO
AZD6244
M C M C
50
37
cjun
FIGURE
S19

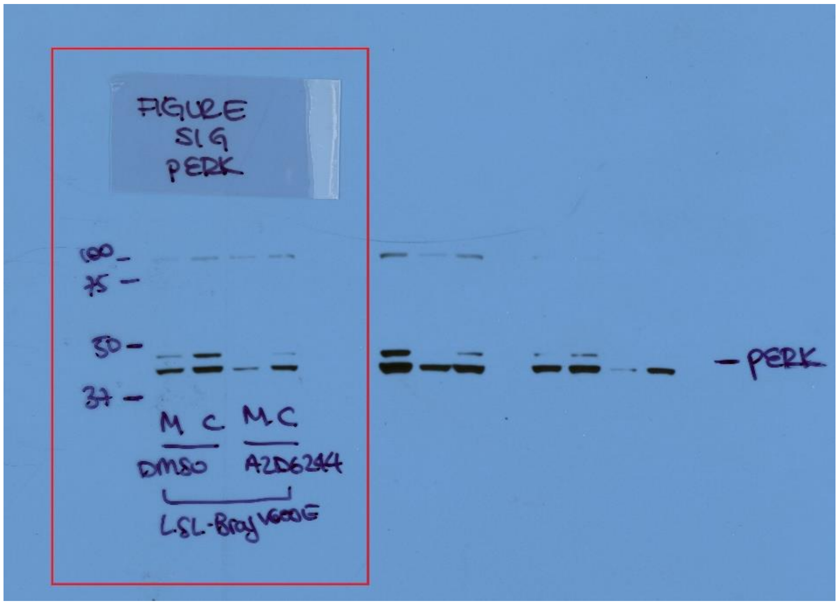
FIGURE
S1G
pERK
100
75
50
37
M C M C
DMSO
AZD6244
LSL-Braf V600E
pERK

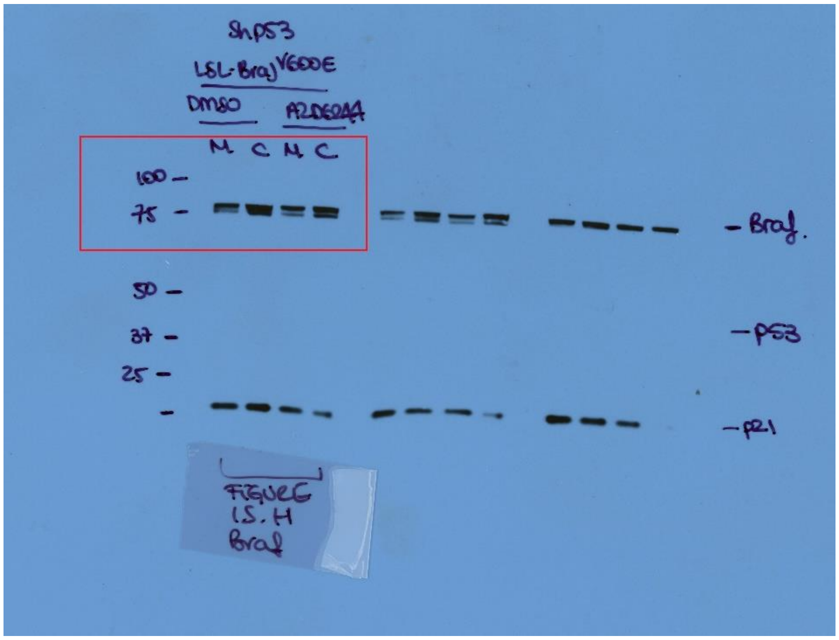
Shp53
LSL-Braf V600E
DMSO
AZD6244
M C M C
100
75
50
37
25
Braf.
p53
p21
FIGURE
1S.H
Braf

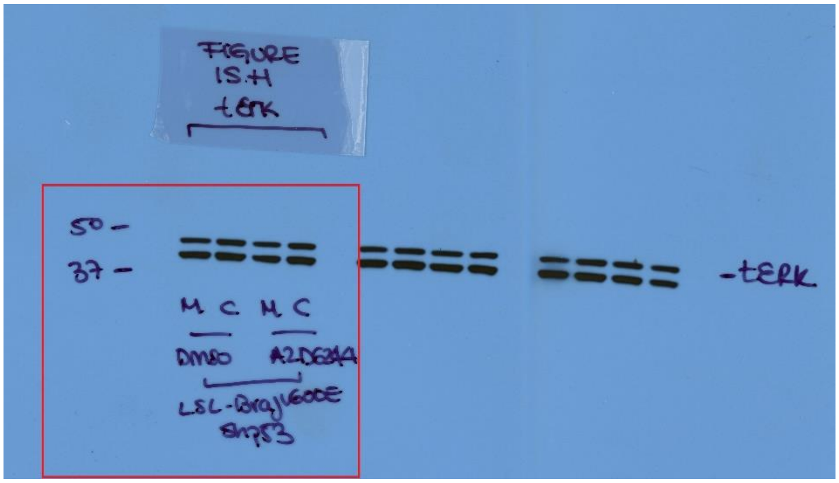
FIGURE
S1H
tERK
50 –
37 –
M C M C
DMSO
AZD6244
-tERK

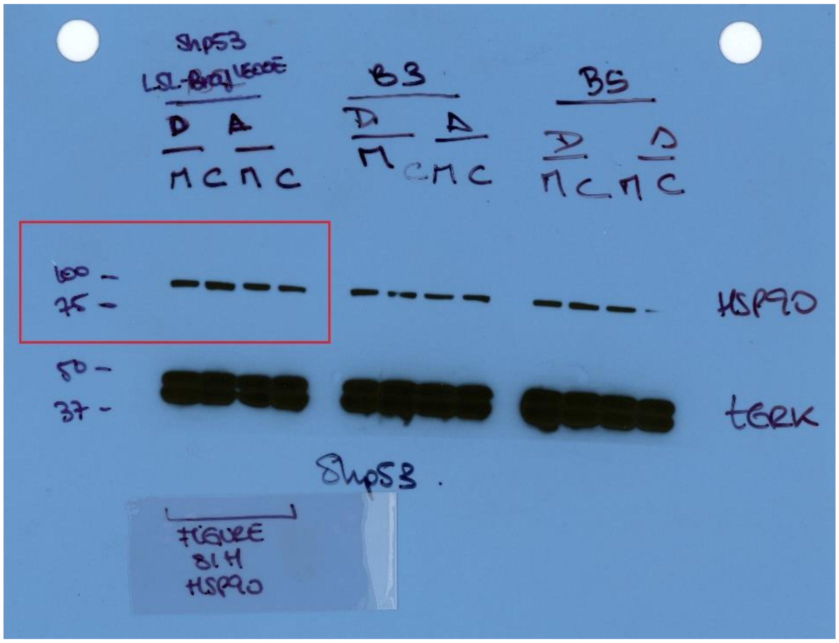
B3
B5
D A
M C M C
100 –
75 –
HSP90
50 –
37 –
tERK
FIGURE
S1H
HSP90

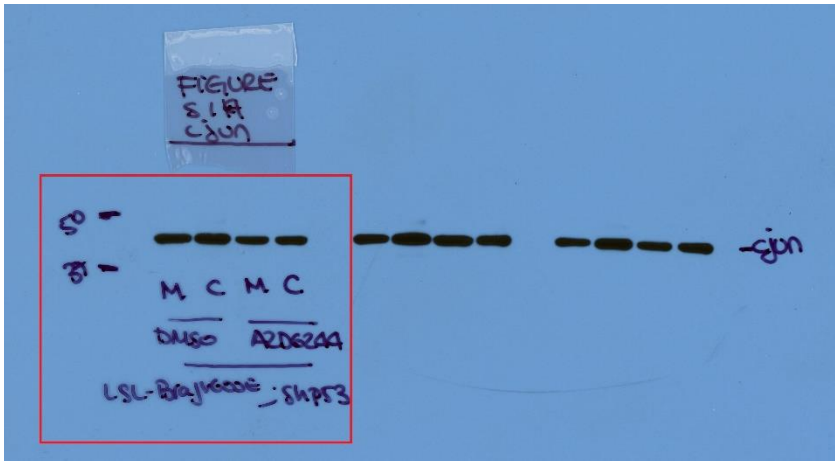
FIGURE
S1H
cJun
50 –
M C M C
DMSO
AZD6244
-cJun

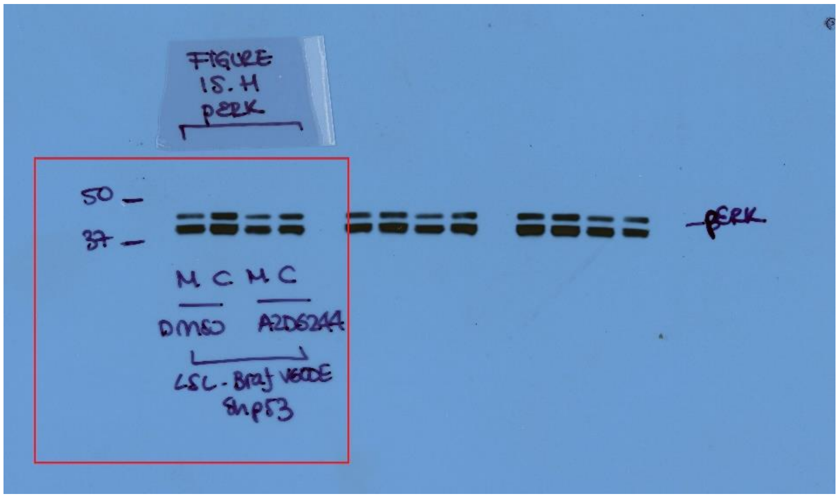
FIGURE
1S.H
pERK
50
37
M C M C
DMSO
AZD6244
LSL-Braf V600E
Shp53
pERK

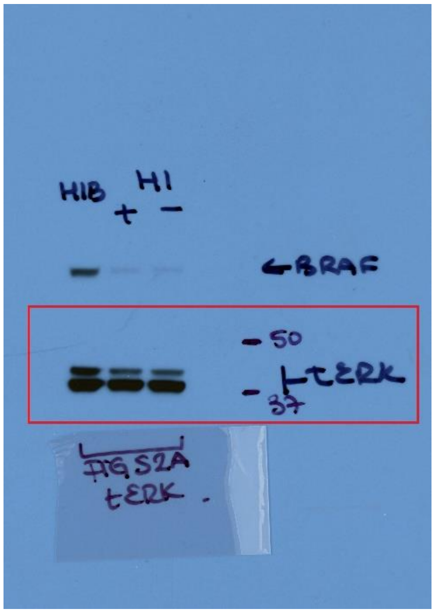
HIB
HI
+
−
BRAF
50
37
tERK
FIGS2A
tERK

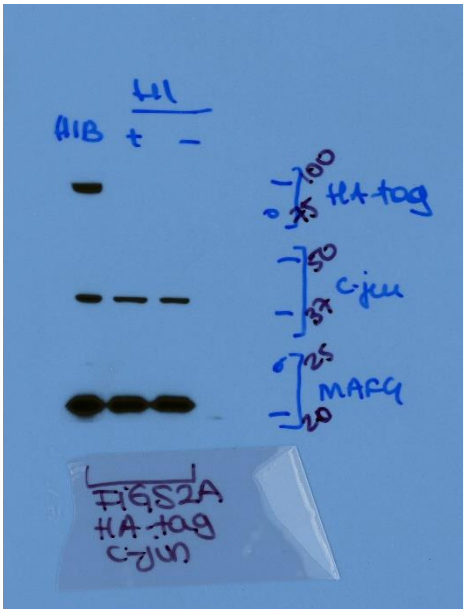
HIB
HI
+
−
100
75
HA-tag
50
37
c-jun
25
20
MAFG
FIGS2A
HA-tag
c-jun

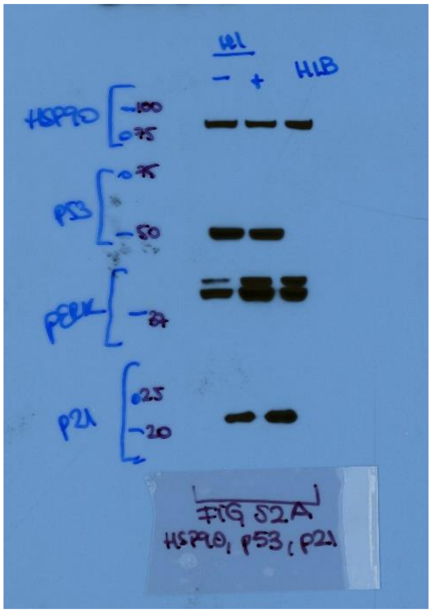
H1
- +
H1B
HSP90
100
75
P53
75
50
pERK
37
P21
25
20
FIG S2A
HSP90, p53, p21

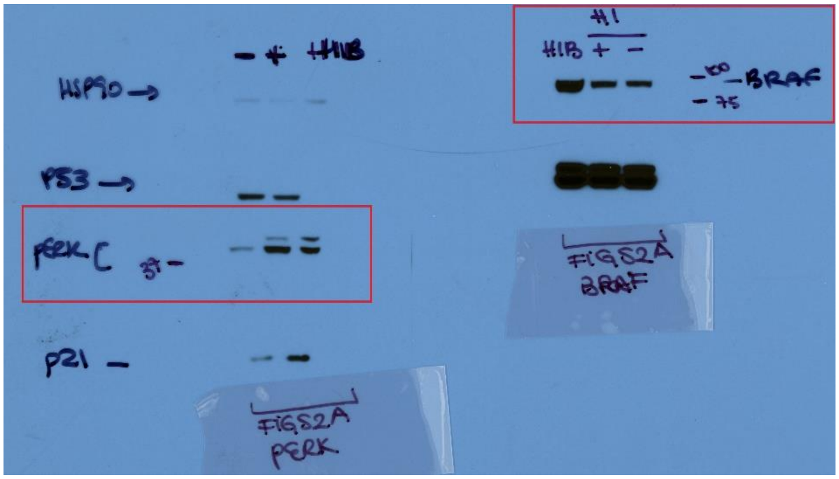
- + H1B
HSP90
P53
pERK
37
p21
FIG S2A
pERK
H1
H1B + -
100
75
BRAF
FIG S2A
BRAF

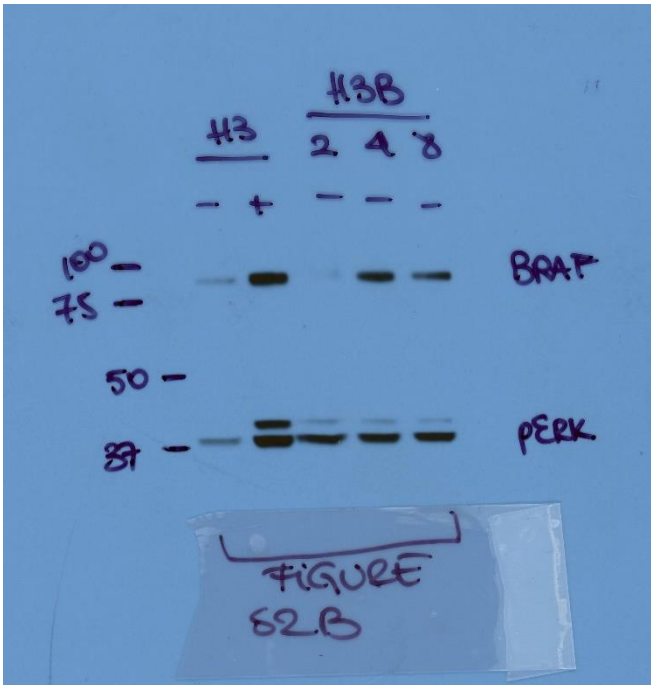
H3
H3B
2 4 8
- + - - -
100
75
BRAF
50
37
pERK
FIGURE
S2B

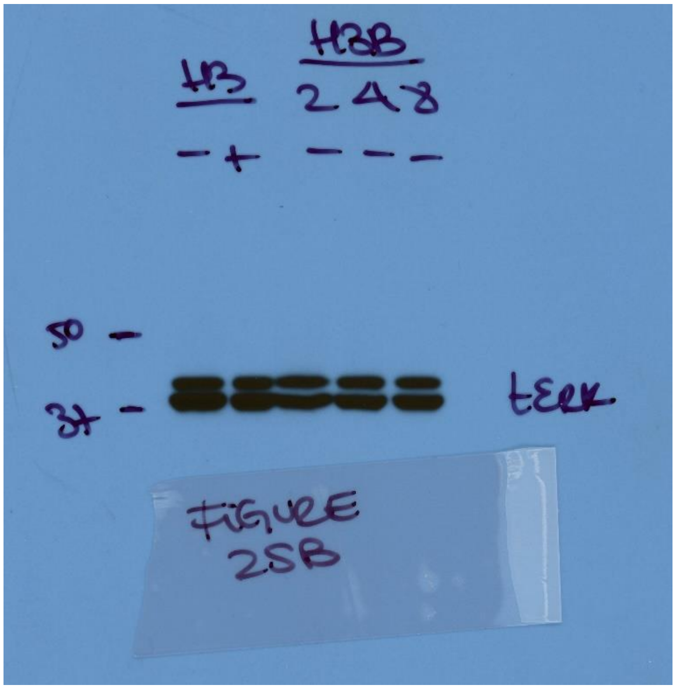
H3
H3B
2 4 8
- + - - -
50
37
tERK
FIGURE
2SB

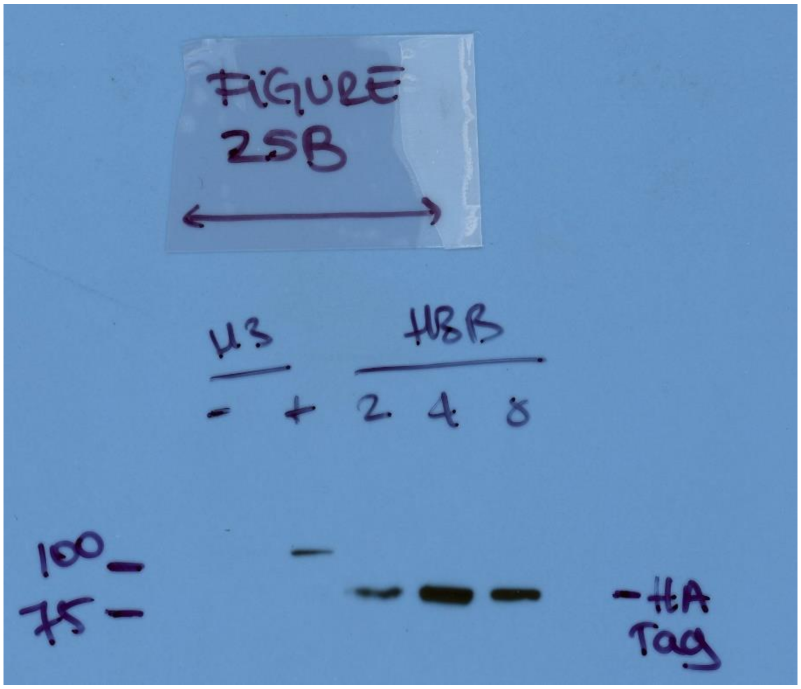

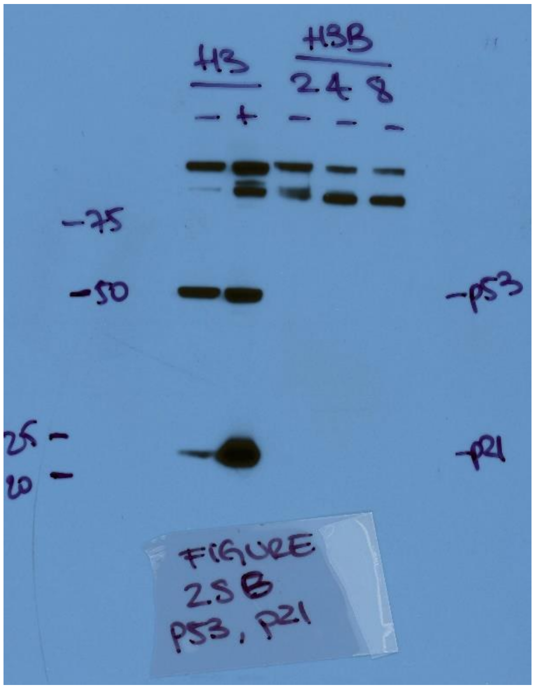

Figure S2B HSP90

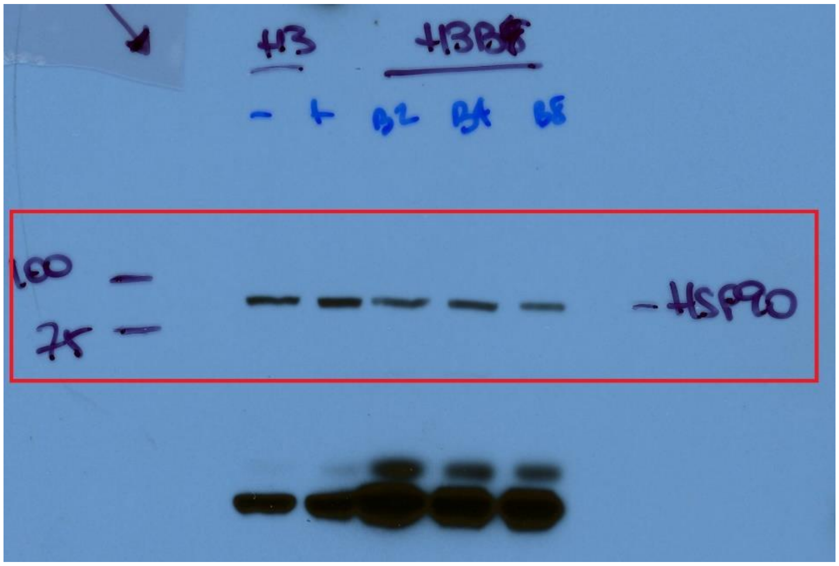

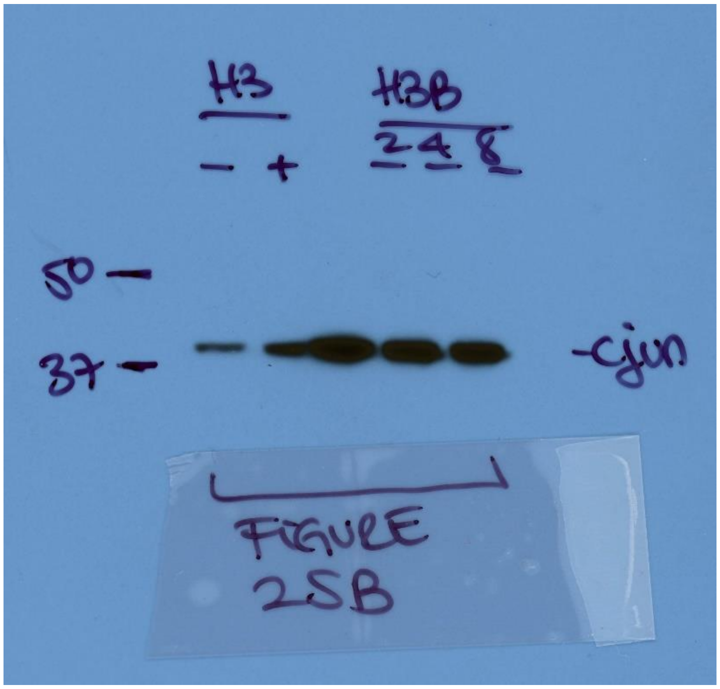
H3
H3B
- +
2 4 8
50
37
cjun
FIGURE
25B

Figure S2D Hermes 1

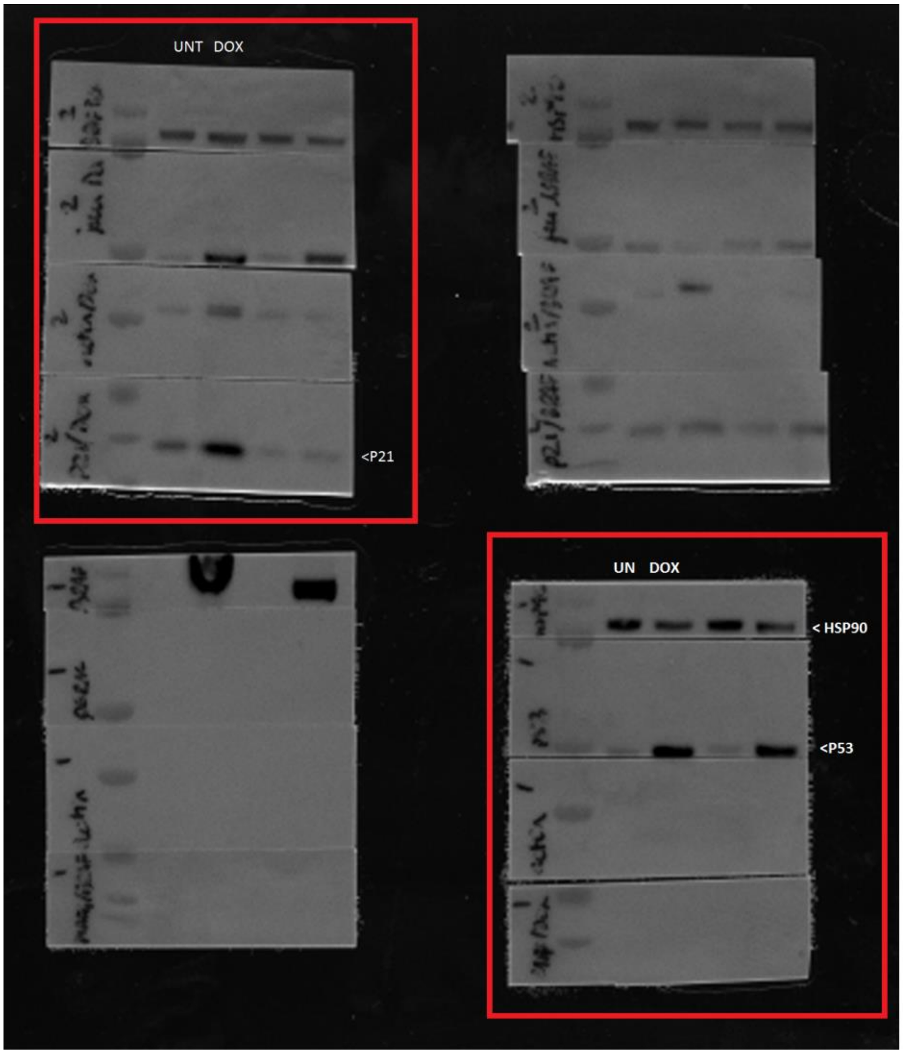

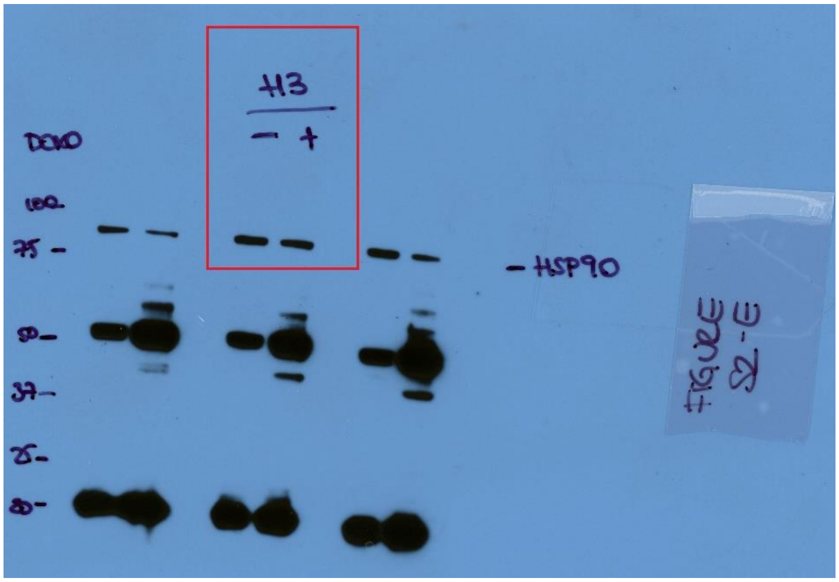

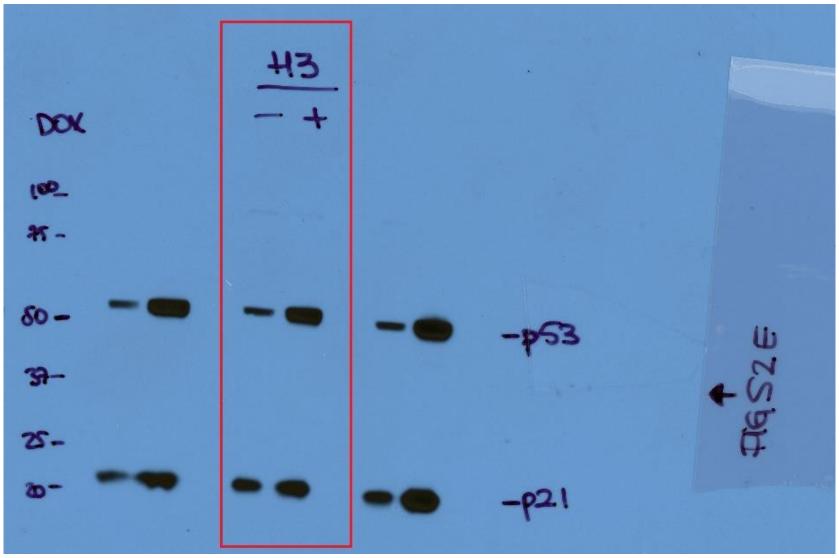
H3
DOX
-p53
-p21
FIG S2E

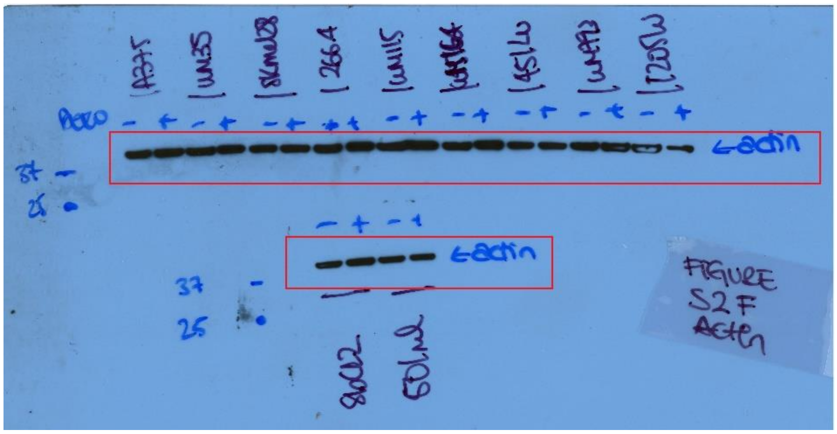
FIGURE
S2F

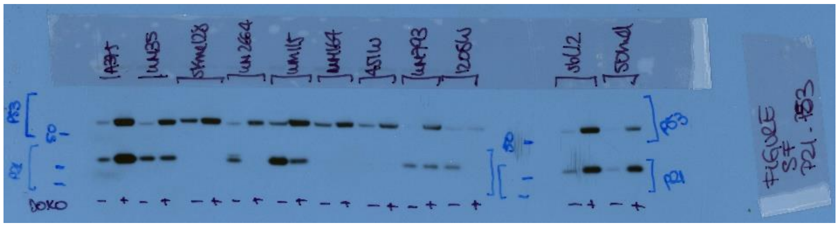
FIGURE
