# Supplementary material for: A MAPK/miR-29 Axis Suppresses Melanoma by Targeting MAFG and MYBL2
Source: Cancers (Basel). 2021 Mar 19;13(6):1408. doi: 10.3390/cancers13061408 (PMC8003541; doi:10.3390/cancers13061408)
Supplement: Supplementary file 1 [file cancers-13-01408-s001.zip › Supplementary Materials.pdf]

cancers

MDPI

Supplementary Materials: A MAPK/miR-29 axis suppresses melanoma by targeting MAFG and MYBL2

Olga Vera, Ilah Bok, Neel Jasani, Koji Nakamura, Xiaonan Xu, Nicol Mecozzi, Ariana Angarita, Kaizhen Wang, Kenneth Y. Tsai, and Florian A. Karreth

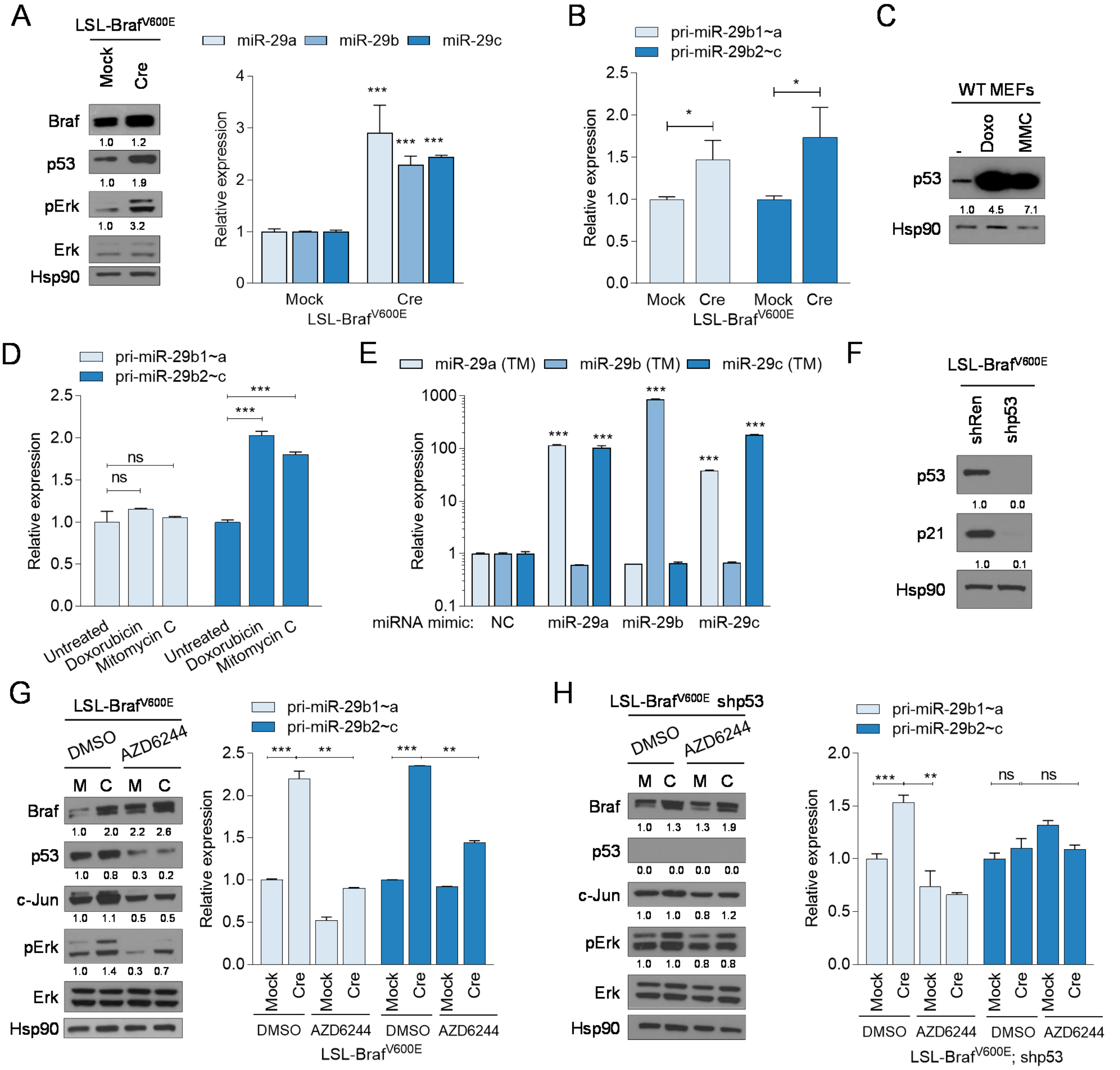

Figure S1. Oncogenic BRAF promotes miR-29 expression in MEFs. (**A**) Induction of Braf^{V600E} expression by Adeno-Cre (Cre) in LSL-Braf^{V600E} MEFs elevates expression of p53 and pERK (Western blot, left panel) and mature miR-29a, miR-29b, and miR-29c (qRT-PCR, right panel). MEFs infected with empty adenovirus (Mock) serve as controls. (**B**) qRT-PCRs showing the expression of pri-miR-29b1~a and pri-miR-29b2~c in LSL-Braf^{V600E} MEFs following Adeno-Cre/Mock infection. (**C**) Effect of Doxorubicin (Doxo) and Mitomycin C (MMC) on p53 in wiltdype MEFs. (**D**) qRT-PCRs showing the expression of pri-miR-29b1~a and pri-miR-29b2~c in wildtype MEFs following treatment with Doxorubicin or Mitomycin C. (**E**) Quantification by qRT-PCR of mature of miR-29a, -29b and -29c after overexpression of microRNA mimics in A375 cells. Cells overexpressing a negative control mimic were used as controls. TM, TaqMan probe. (**F**) Confirmation of p53 silencing in LSL- Braf^{V600E} MEFs infected with shp53 retrovirus. (**G**,**H**) Effect of MEK inhibitor (AZD6244) on the MAPK pathway and pri-miR-29b1~a and pri-miR-29b2~c expression in LSL-Braf^{V600E} (**G**) and shp53-expressing LSL-Braf^{V600E} MEFs (**H**). For all

Western blots, Hsp90 was used as loading control. The mean ± SEM of one representative out of two independent experiments performed in triplicate in three different cell lines is shown. Gene expression levels are normalized to U6 (mature miR-29) or b-Actin (pri-miR-29) as endogenous control. All western blots show the intensity ratio of the protein of interest normalized to HSP90 or Actin. ns, not significant; * $p < 0.05$; ** $p < 0.01$; *** $p < 0.001$.

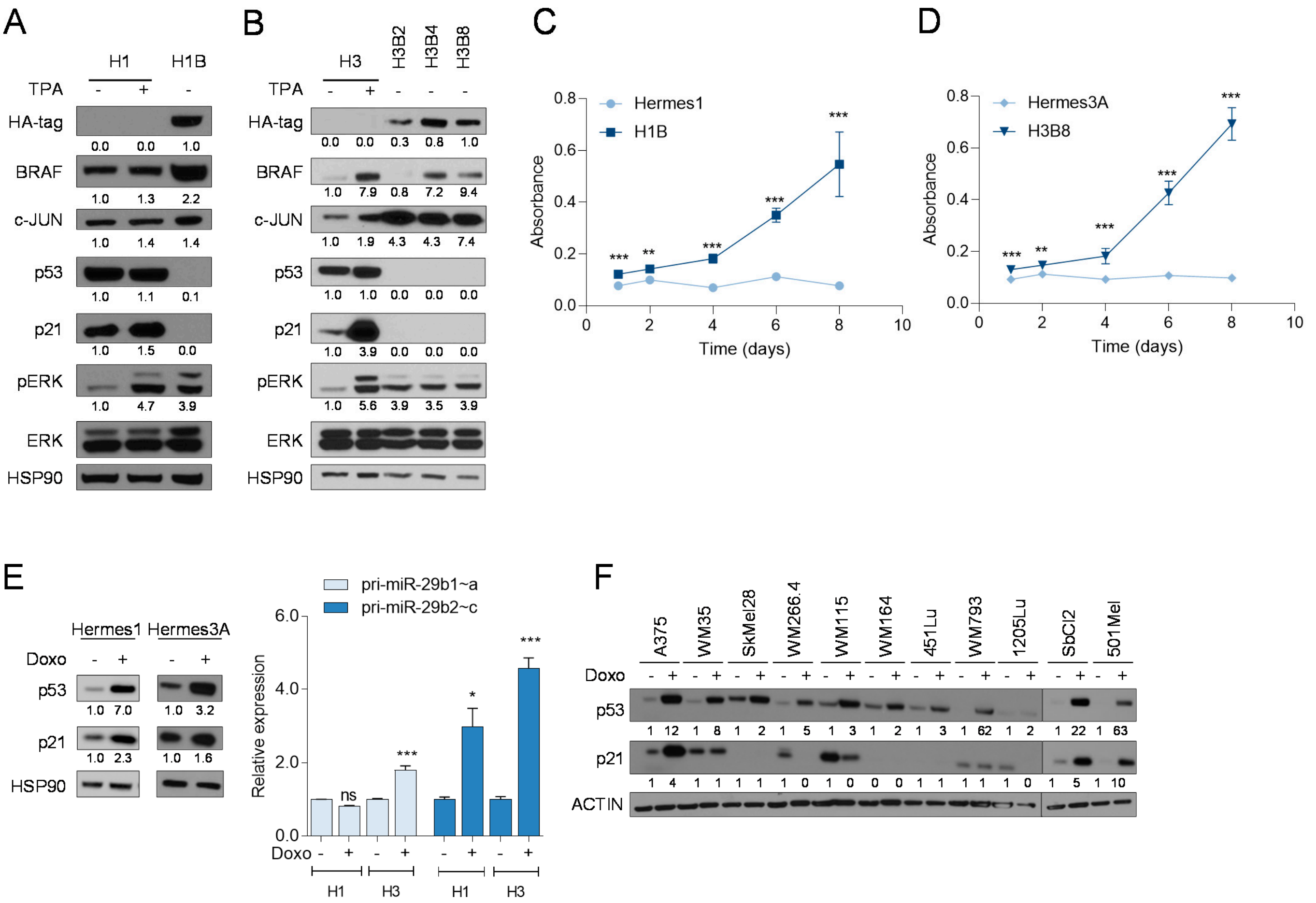

Figure S2. p53 activity is impaired in BRAF-mutant melanocytes and melanoma cell lines. (**A**,**B**) Comparison of protein expression between parental melanocytes Hermes1 (H1, A) and Hermes3A (H3, B) cultured with (+) and without TPA (−) and BRAF^{V600E} melanocytes (H1B, H3Bs) in absence of TPA (−). (**C**,**D**) Proliferation assay comparing Hermes1 with H1B (**C**) and Hermes3A with H3B8 (**D**) cell lines cultured in absence of TPA. The combined mean ± SEM of two independent experiments performed in quadruplicate is shown. (**E**) Effect of Doxorubicin (Doxo) on pri-miR-29b1~a and pri-miR-29b2~c expression in human melanocytes Hermes1 (H1) and Hermes3A (H3). (**F**) Effect of Doxorubicin (Doxo) on p53 and p21 in a panel of melanoma cell lines. All western blots show the intensity ratio of the protein of interest normalized to HSP90 or Actin. Ns, not significant; * $p < 0.05$; ** $p < 0.01$; *** $p < 0.001$.

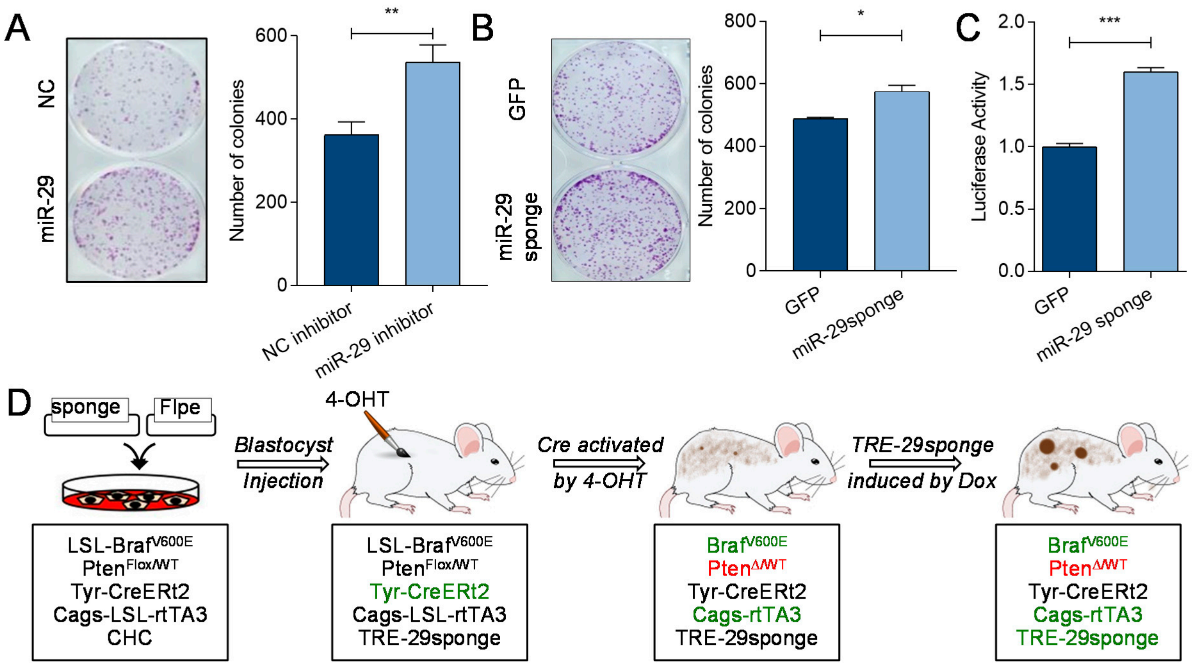

Figure S3. Inactivation of miR-29 promotes melanoma. (A) Effect of miR29 inactivation by hairpin inhibitors on colony formation of A375 cells. (B) Effect of miR-29 inactivation by a miR-29 sponge construct on colony formation of A375 cells. The mean ± SEM of one representative out of two independent experiments performed in triplicate is shown in (A) and (B). (C) Effect of the miR-29 sponge on miR-29-Luciferase reporter activity in A375 cells. The combined mean ± SEM of three independent experiments performed in quadruplicate is shown. (D) Outline of the embryonic stem cell-genetically engineered mouse model approach [21] where a Dox-inducible miR-29 sponge is expressed in $Braf^{V600E}$; $Pten^{\Delta/WT}$ melanocytes. * $p < 0.05$; ** $p < 0.01$; *** $p < 0.001$.

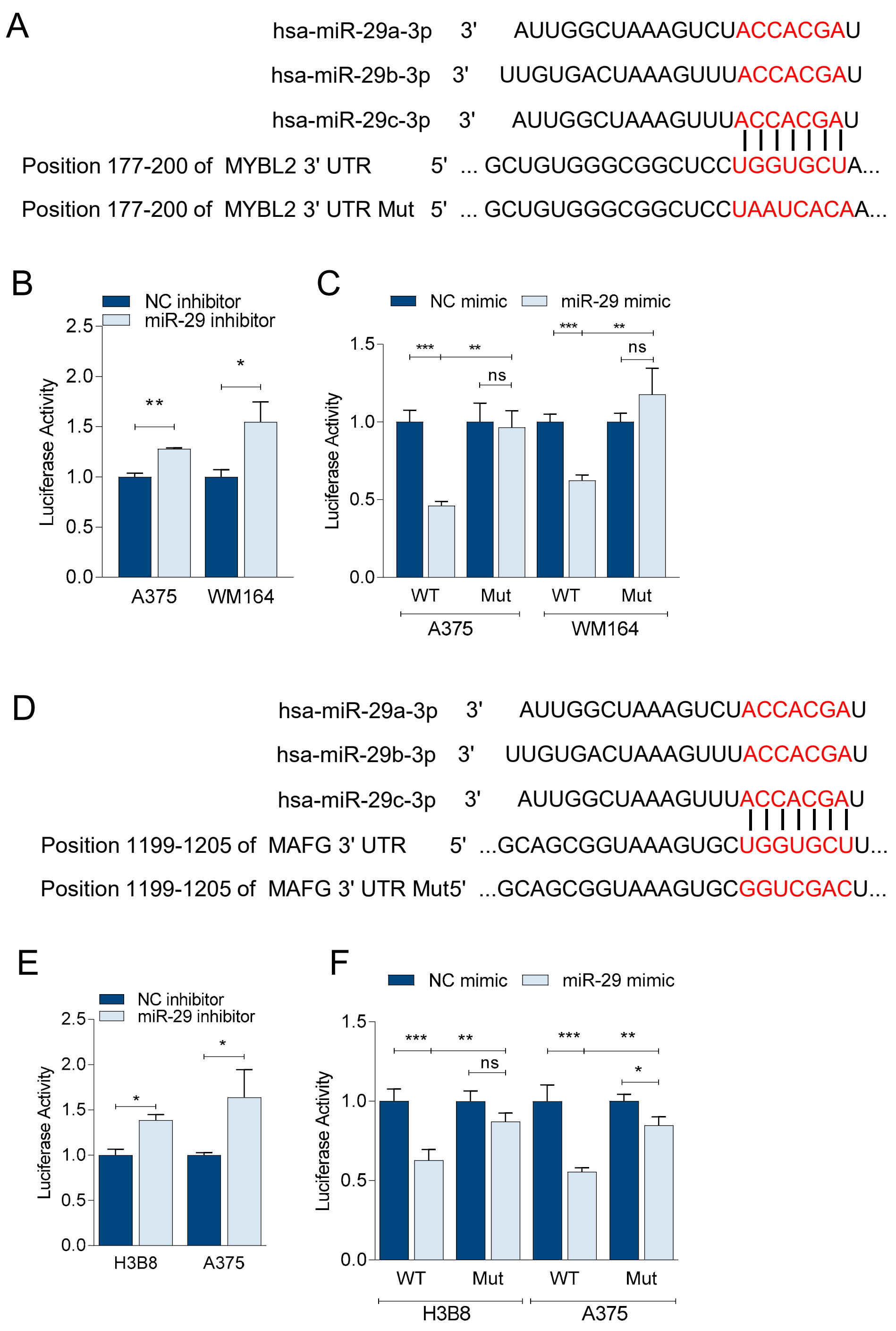

Figure S4. MAFG and MYBL2 are direct targets of miR-29. (**A**) Alignment of mature miR-29a, -29b and -29c with the miR-29 binding site in the human MYBL2 3′UTR. The mutated miR-29 binding site used in the Luciferase reporter assays is shown below. (**B**) Activity of MYBL2 3′UTR Luciferase reporter in response to miR-29 inhibitors. (**C**) Activity of MYLB2 wildtype or miR-29 binding site-mutant MYBL2 3′UTR Luciferase reporter in response to miR-29 mimics. (**D**) Alignment of mature miR-29a, -29b and -29c with the miR-29 binding site in the human MAFB 3′UTR. The mutated miR-29 binding site used in the Luciferase reporter assays is shown below. (**E**) Activity of MAFG 3′UTR Luciferase reporter in response to miR-29 inhibitors. (**F**) Activity of MAFG wildtype or miR-29 binding site-mutant MAFG 3′UTR Luciferase reporter in response to miR-29 mimics. The combined mean ± SEM of two independent experiments performed in quadruplicate is shown. ns, not significant; * $p < 0.05$; ** $p < 0.01$; *** $p < 0.001$.

Table S1. Summary of the mutation status for BRAF, NRAS and P53 of the melanoma cell lines used in this study.

Cell Line	Mutations	p53 Induction in Response to Doxorubicin	p21 Induction in Tesponse to Doxorubicin
A375	BRAF: V600E<br>NRAS: WT<br>p53: unknown	+++	++
WM35	BRAF: V600E<br>NRAS: WT<br>p53: unknown	++	=
SK-Mel-28	BRAF: V600E<br>NRAS: WT<br>p53: L145R	=	=
WM266-4	BRAF: V600E<br>NRAS: WT<br>p53: unknown	+	=
WM-115	BRAF: V600E<br>NRAS: WT<br>p53: WT	+	=
WM164	BRAF: V600E<br>NRAS: WT<br>p53: Y220C	=	=
451Lu	BRAF: V600E<br>NRAS: WT<br>p53: Y220C	=	=
WM793	BRAF: V600E<br>NRAS: WT<br>p53: WT	+++	=
1205Lu	BRAF: V600E<br>NRAS: WT<br>p53: WT	=	=
SbCl2	BRAF: WT<br>NRAS: Q61K<br>p53: unknown	+++	++
501Mel	BRAF: V600E<br>NRAS: WT<br>p53: WT	+++	++

p53 and p21 induction columns summarize results observed in Supplementary Figure S2F. =, no change; +, low induction; ++, moderate induction, +++ high induction.

Table S2. List of primers and reagents used in this manuscript.

SYBER-Green Primers	Sequence
m_Mybl2_Fwd	ATCCACTCCAGTGTGTAGCCAAA
m_Mybl2_Rvs	CCATGGAGTACTTCTGATCTGGG
m_Scl31A1_Fwd	GGGGCTTACCCTGTGAAGACTTT
m_Scl31A1_Rvs	CGTCCGTGTGGTTCATACCC
m_Tubb2a_Fwd	ATCGGCGCTAAGTTTTGGGA
m_Tubb2a_Rvs	CAAGTCACTGTCGCCATGGTAA
m_Kctd5_Fwd	GATTCGGACAAGGATGAAAC
m_Kctd5_Rvs	CTTCCTCTGCGAGGTCTTTG
m_Sh3bp5l_Fwd	ATCCTAGGATACAGGAGGAG
m_Sh3bp5l_Rvs	TCAATACAGCTCCCCAAGTG
m_Sms_Fwd	AGGAGACTGCTATCAGGTTC
m_Sms_Rvs	AGGTCGAGAATCAGTCTGAG
m_Rcc2_Fwd	GCCATGGGCTATTCACACTC
m_Rcc2_Rvs	TCAGGTAGCCTCTGCAGC
m_Nckap5l_Fwd	TACTGAACCAGAAGGACCTG
m_Nckap5l_Rvs	TGGTCCCTCAGAGACATTTG
m_Mafg_Fwd	GACCCCCAATAAAGGAAACAA
m_Mafg_Rvs	TCAACTCTCGCACCGACAT
m_Actin_Fwd	TTGCTGACAGGATGCAGAAG
m_Actin_Rvs	ACATCTGCTGGAAGGTGGAC
MYBL2_FWD	CATTGTGGATGAGGATGTGAAGC
MYBL2_RVS	TGGTTGAGCAAGCTGTTGTCTTC
SCL31A1_FWD	GGGGATGAGCTATATGGACTCC
SCL31A1_RVS	TCACCAAACCGGAAAACAGTAG
TUBB2A_FWD	ACAATGAGGCTGCTGGTAACA
TUBB2A_RVS	TCTGACTCCTTCCTCACCAC
KCTD5_FWD	AAGTTCGAGCAGTTGGTCAG
KCTD5_RVS	GCAAAATCTTGGCCTTCTCG
SH3BP5L_FWD	ACCTGCAAGGTGAATGAGGC
SH3BP5L_RVS	TTGTGCTCCTCCAGGATCTG
SMS_FWD	ACAGCTGTTCCAATCTCCAC
SMS_RVS	CGACAGTGCTTCTGTCAGAT
RCC2_FWD	TTCCTTTGGGTGCCCTGAA
RCC2_RVS	GGCAGAATCTGTCCATCTTTCG
NCKAP5L_FWD	TTCAGCAGAAACTCCAGCTC
NCKAP5L_RVS	CTCCCAACATACCTCTCTCT
MAFG_FWD	CACCCTTCTCTCTTCCCTGCAA
MAFG_RVS	TCTCTCTCCCGCAACTCTCTCT
MAFF_FWD	ATCCCCTATCCAGCAAAGCTC
MAFF_RVS	TTGAGCCGTGTCACCTCCTC
MAFK_FWD	CGACTAATCCCAAACCGAAT
MAFK_RVS	ACATGGACACCAGCTCATCA
DNMT3B_FWD	AGTCGAAGGTGCGTCGTG
DNMT3B_RVS	AGCCATTGTTCTCGGCTCT
AKT3_FWD	GAGGACCGCACACGTTTCTA
AKT3-RVS	TGTCTTCATGGTGGCTGCAT
MCL1-FWD	GAGGACGAGTTGTACCGGCAG
MCL1_RVS	CGTTTTTGATGTCCAGTTTCCGA
GAPDH_FWD	GAGAGACCCTCACTGCTG
GAPDH_RVS	GATGGTACATGACAAGGTGC

Cloning Primers	Sequence
MAFG-pLEGB_FWD	acagtctagaggatcctcgactggatccggtacc
MAFG-pLEGB_RVS	gaggttgattgtcgacttgggcatggccaggtagc

3'UTR-MAFG-pSICHECK2_FWD	taggcgatcgctcgagcgatcgccaaagtccaag
3'UTR-MAFG-pSICHECK2_RVS	tgtatatgatcagcggaccgggccgctggccgcaa
psicheck2-miR-29 reporter_FWD	tcgacTAACCGATTTCAcGATGGTGCTAgc
psicheck2-miR-29 reporter_RVS	ggccgcTAGCACCATCgTGAAATCGGTTAg
3'UTR-MYBL2-pSICHECK2_FWD	TAGGCGATCGCTCGAGGGTGTTGAGGGTGTCAC-GAG
3'UTR-MYBL2-pSICHECK2_RVS	TTGCGGCCAGCGGCCGCAAGAGA-GAGGCAATTTTATTCTTCCAAAAAAATGC
SDM_MAFG_FWD	gactctggtgaCCTTTGACCTGTGGGTGTC
SDM_MAFG_RVS	gaccgcactttaCCGCTGCACAAAACCTCA
SDM_MYBL2_FWD	CACAACAACAAAGTTCCACTTCCAGGTCTGC
SDM_MYBL2_RVS	ATTAGGAGCCGCCCACAGCTGG

**Dharmacon siRNAs**	**Catalog #**
ON-TARGETplus Human NCKAP5L siRNA-SMARTPOOL-5nmol	L-026953-03-0005
ON-TARGETplus Human SMS siRNA-SMARTPOOL-5nmol	L-017273-00-0005
ON-TARGETplus Human SH3BP5L siRNA-SMARTPOOL-5nmol	L-014642-02-0005
ON-TARGETplus Human TUBB2A siRNA-SMARTPOOL-5nmol	L-008260-00-0005
ON-TARGETplus Human RCC2 siRNA-SMARTPOOL-5nmol	L-013835-00-0005
ON-TARGETplus Human SLC31A1 siRNA-SMARTPOOL-5nmol	L-007531-02-0005
ON-TARGETplus Human MAFK siRNA-SMARTPOOL-5nmol	L-008580-00-0005
ON-TARGETplus Human MAFF siRNA-SMARTPOOL-5nmol	L-003903-00-0005
ON-TARGETplus Human KCTD5 siRNA-SMARTPOOL-5nmol	L-021199-00-0005
ON-TARGETplus Human MYBL2 siRNA-SMARTPOOL-5nmol	L-010444-00-0005
SMARTpool: ON-TARGETplus MAFG siRNA	L-009109-00-0005
ON-TARGETplus Non-targeting Pool	D-001810-10-05

**Mimics and Inhibitors**	**Catalog #**
miRIDIAN microRNA mmu-miR-29a-3p hairpin inhibitor	IH-310521-08-0002
miRIDIAN microRNA mmu-miR-29b-3p hairpin inhibitor	IH-310381-07-0002
miRIDIAN microRNA mmu-miR-29c-3p hairpin inhibitor	IH-310522-08-0002
miRIDIAN microRNA Hairpin Inhibitor Negative Control #1	IN-001005-01-05
miRIDIAN microRNA mmu-miR-29a-3p mimic	C-310521-07-0002
miRIDIAN microRNA mmu-miR-29b-3p mimic	C-310381-05-0002
miRIDIAN microRNA mmu-miR-29c-3p mimic	C-310522-07-0002
miRIDIAN microRNA Mimic Negative Control #1	CN-001000-01-05

Data S1: Supplementary Methods

1. Cell Culture and Treatments

The human immortalized melanocytes cell lines Hermes1, Hermes2, Hermes3A, and Hermes4B were obtained from the Functional Genomics Cell Bank at St George's, University of London, UK, and cultured in RPMI media supplemented with 10% FBS, 10 ng/mL hSCF (R&D, Minneapolis, MN, USA Cat # 255-SC), 200 nM TPA (Sigma, St. Louis, MO, USA Cat # P8139), 200pM Cholera Toxin (Sigma, Cat # C8052), and 10 nM Endothelin-1 (Sigma, Cat # E7764) at 37 °C in a humidified atmosphere containing 10% CO_2. A375 and SK-Mel28 cells were purchased from ATCC (Manassas, VA, USA) WM164, WM35, WM793 and 1205Lu, WM115, WM266.4, 451Lu cells were a gift from Meehard Herlyn, Wistar Institute, Philadelphia, PA, USA from the Wistar Collection of Melanoma cell lines, SbCl2 were provided by David Tuveson, Cold Spring Harbor Laboratory, Cold Spring Harbor, NY, USA, and 501Mel were from Keiran Smalley (Departments of Tumor Biology, H. Lee Moffitt Cancer Center and Research Institute, Tampa, FL, USA). All cancer cell lines were cultured in RPMI containing 5% FBS at 37 °C in a humidified atmosphere containing 5% CO_2. The mutation status of the melanoma cell lines used in the study is detailed in Table S1. The $BRAF^{V600E}$-expressing human melanocyte cell lines were derived by infecting Hermes1 and Hermes3A with a $BRAF^{V600E}$-pLenti-Hygro (provided by Lixin Wan, Departments of Molecular Oncology, H. Lee Moffitt Cancer Center and Research Institute, Tampa, USA) in the presence of 8 μg/mL Polybrene. Transduced cells were selected in 100 μg/mL of Hygromycin (Invivogen, San Diego, CA, USA Cat # ant-hg-1) for seven days in the absence of TPA. Four independent clones were picked and expanded until stable cell lines were obtained. MEFs were generated from E13.5-E14.5 embryos from LSL-$Braf^{V600E}$ [61] or LSL-$Kras^{G12D}$ mice [62] and cultured in DMEM containing 10% FBS at 37 °C in a humidified atmosphere containing 5% CO_2. Wildtype MEFs were derived from littermate embryos that did not harbor the LSL-$Braf^{V600E}$ or LSL-$Kras^{G12D}$ alleles. LSL-$Braf^{V600E}$ primary mouse melanocytes (PMM) were isolated as previously described [59] with minor modifications. To recombine floxed alleles, MEFs and PMM were infected with approximately 10^7 pfu/mL Ad5CMVCre or Ad5CMVempty adenovirus obtained from the University of Iowa Viral Vector Core. Lenti-X 293T cells were obtained from Takara Bio (Mountain View, CA, USA) and cultured in DMEM containing 10% FBS at 37 °C in a humidified atmosphere containing 5% CO_2. All cell lines were routinely tested for mycoplasma using MycoAlert Plus (Lonza, Basel, Switzerland, Cat # LT07-710), and human melanoma cell lines were STR authenticated by Moffitt's Molecular Genomics Core. Doxorubicin (Fisher Scientific, Waltham, MA, USA, Cat # BP25131) was used at a final concentration of 10 μM for 24 h and AZD6244 (Selleckchem, Houston, TX, USA, Cat # S1008) was used at a final concentration of 0.5 μM for 8 or 24 h.

2. RNA Isolation and Quantitative RT-PCR

Total RNA was isolated using TRI-Reagent (Zymo Research, Irvine, CA, USA, Cat # R2050-1-200) and mature miRNAs were isolated using the miRNeasy Mini Kit (Qiagen, Germantown, MD, USA, Cat # 217004) according to the manufacturers' recommendations. For qRT-PCR, 500 ng of total RNA were retrotranscribed using PrimeScript RT Master Mix (Takara Bio, Cat. # RR036A), and subsequent TaqMan assay-based or SYBR Green-based qPCRs were performed using PerfeCTa qPCR ToughMix (QuantaBio, Beverly, MA, USA, Cat. # 97065-960) or PerfeCTa SYBR Green FastMix (QuantaBio, Cat. # 95073-012), respectively. Mature miRNAs were retrotranscribed using TaqMan MicroRNA Reverse Transcription Kit (Fisher Scientific, Waltham, MA, USA, Cat # 4366596) and analyzed by qPCR using PerfeCTa qPCR ToughMix (QuantaBio, Cat. # 97065-960). Samples were analyzed in triplicate using the StepOne Plus PCR system (Applied Biosystems, Foster City, CA, USA). The comparative threshold cycle method (2-ΔΔCt) was used to calculate the relative expression levels. snoU6 was used as endogenous control for mature miRNAs while GAPDH or β-Actin were used for mRNAs and pri-miRNAs. TaqMan Probes for expression analyses were purchased from Thermo Fisher Scientific (U6 snRNA: 001973;

mouse β-actin: Mm02619580_g1; human β-ACTIN: mouse Cdkn1a: Mm04205640_g1; mmu-mir-29a: Mm03306859_pri; mmu-mir-29b-2: Mm03307196_pri; mmu-mir-29c: Mm03306860; mmu-mir-29b-1; Mm03306189_pri; hsa-mir-29a: Hs03302672_pri; hsa-mir-29c: Hs04225365_pri; hsa-miR-29a: 002112; hsa-miR-29b: 000413; hsa-miR-29c: 000587). Primers for SYBR Green qPCR are listed in Table S2.

3. RNA-sequencing

Total RNA from cells was isolated using miRNeasy Mini Kit (Qiagen, Germantown, MD, USA, Cat # 217004) and RIN were analyzed on an Agilent TapeStation (Agilent, Santa Clara, CA, USA). Samples were sent to Novogene (Sacramento, CA, USA) for sequencing. Library preparation was performed by NEBNext® Ultra™ RNA Library Prep Kit for Illumina® (Illumina, San Diego, CA, USA) which is for non-stranded libraries. Sequencing was performed on the NovaSeq 6000 (Illumina, San Diego, CA, USA) with a paired end 150 BP method. Downstream analysis was performed using a combination of programs including STAR, HTseq, Cufflink and our wrapped scripts. Alignments were parsed using Tophat program (https://tophat.com/, accessed on 17 March 2021) and differential expressions were determined through DESeq2/edgeR. GO and KEGG enrichment were implemented by the ClusterProfiler (https://bioconductor.org/packages/release/bioc/html/clusterProfiler.html, accessed on 17 March 2021). Gene fusion and difference of alternative splicing event were detected by Star-fusion (https://github.com/STAR-Fusion/STAR-Fusion/wiki, accessed on 17 March 2021) and rMATS software (http://rnaseq-mats.sourceforge.net/, accessed on 17 March 2021). Reference genome and gene model annotation files were downloaded from genome website browser (NCBI (https://www.ncbi.nlm.nih.gov/, accessed on 17 March 2021)/UCSC (https://genome.ucsc.edu/, accessed on 17 March 2021)/Ensembl (https://useast.ensembl.org/index.html), accessed on 17 March 2021)) directly. Indexes of the reference genome was built using STAR and paired-end clean reads were aligned to the reference genome using STAR (v2.5). STAR used the method of Maximal Mappable Prefix (MMP) which can generate a precise mapping result for junction reads. HTSeq v0.6.1 was used to count the read numbers mapped of each gene. And then FPKM of each gene was calculated based on the length of the gene and reads count mapped to this gene. FPKM, Reads Per Kilobase of exon model per Million mapped reads, considers the effect of sequencing depth and gene length for the reads count at the same time, and is currently the most commonly used method for estimating gene expression levels [63]. Differential expression analysis between was performed using the DESeq2 R package (v2_1.6.3). DESeq2 provide statistical routines for determining differential expression in digital gene expression data using a model based on the negative binomial distribution. The resulting *p*-values were adjusted using the Benjamini and Hochberg's approach for controlling the False Discovery Rate (FDR). Genes with an adjusted *p*-value < 0.05 found by DESeq2 were assigned as differentially expressed. Accession number: PRJNA624657 (https://www.ncbi.nlm.nih.gov/sra; accessed on 15 March 2021).

4. Plasmids

pBabe, pBabe-Braf^{V600E}, and pBabe-Kras^{G12D} were gifts from David Tuveson. pLenti-GFP-puro was purchased from Addgene (Watertown, MA, USA plasmid #17448). The CMV promoter and puromycin in pLenti-GFP-puro were replaced with the EF1α promoter and blasticidin, respectively, using standard In-Fusion cloning (Takara Bio, Cat. # 638911) to create pLEGB. The Myc-DDK-tagged ORF clone of MAFG (RC221486, OriGene Rockville, MD, USA) was a gift from Inmaculada Ibanez de Caceres (Epigenetics laboratory, INGEMM, Hospital La PAZ, 28046 Madrid, Spain) and cloned into pLEGB to replace GFP. The full length MAFG 3′UTR sequence (NM_002359.3 OriGene, USA) was a gift from Inmaculada Ibanez de Caceres and cloned into psiCHECK2 plasmid by In-Fusion cloning. 391 nucleotides of the human MYBL2 3′UTR sequence were amplified and cloned into psiCHECK2 plasmid by In-Fusion cloning. The psiCHECK2-miR29 Luciferase reporter was created by oligo cloning into psiCHECK2. For inducible expression, MAFG was

cloned into the Dox-inducible pRRL-Blast vector by In-Fusion cloning. All primers for cloning are detailed in Table S2.

5. Site-directed Mutagenesis

We used either the psiCHECK2-MAFG-3′-UTR or psiCHECK2-MYBL2-3′-UTR to generate the miR-29 binding site mutant. For MAFG, four different miR-29 binding sites were predicted by TargetScan, one of which is highly conserved, while only one was identified for MYBL2. Site-directed mutagenesis was performed using the Q5 Site-Directed Mutagenesis Kit (NEB, Ipswich, MA, USA, E0554S) according to the manufacturer's instructions. Primers for site-directed mutagenesis are listed in Table S2.

6. Cell Transfection and Lentiviral Transduction

For miR-29 overexpression and inhibition, 100,000 cells/well were plated in 6-well plates and transfected with 25 to 150 nM of Dharmacon miRIDIAN microRNA miR-29a, miR-29b or miR-29c mimic (C-310521-07-0002, C-310381-05-0002 or C-310522-05-0002), hairpin inhibitor (IH-310521-08-0002, IH-310381-07-0002 or IH-310522-08-0002), or negative controls (CN-002000-01-05; IN-001005-01-05) using JetPrime (VWR, Radnor, PA, USA, Cat # 89129-924) according to the manufacturer's protocol and assayed after 48 h. For Luciferase assays, cells were plated in 96-well plates at a density of 10,000 cells/well. Either MAFG or MYBL2 psiCHECK2-3′UTR_wildtype or psiCHECK2-3′UTR_miR-29-mutant were co-transfected with miR-29 mimics or inhibitors, following the procedure described above. Luminescence was assayed after 24 h using the Dual Luciferase Assay System (Promega, Madison, WI, USA, Cat # E1960), according to the manufacturer's instructions. Similarly, miR-29-sponge melanoma cell lines were plated at a density of 10,000 cells/well and transfected with psiCHECK2 or psiCHECK2-miR-29 reporter following the same procedure. Luminescence was assayed after 48 h using the Dual Luciferase Assay System. Results were normalized to Firefly luminescence. For retroviral transductions, Lenti-X 293T cells were transfected with the retroviral vector and Eco helper plasmid at a 2:1 ratio. For lentiviral transductions, Lenti-X 293T cells were transfected with the lentiviral vector and the Δ8.2 and pMD2-VSV-G helper plasmids at a 9:8:1 ratio. Supernatants were collected 48 h after transfection and filtered through a 0.45 μm filter. Cells were plated in 6-well plates at a density of 300,000 cells/well and transduced with supernatants in the presence of 8 μg/mL polybrene for 6 h. Selection was carried out by treating cells with 10 μg/mL Blasticidin for 5 days or 1 μg/mL Puromycin for 4 days. For siRNA transfections, 100,000 cells/well were plated in 6-well plates and transfected with 25 nM of ON-TARGETplus siRNA pool or Non-Targeting control using JetPrime according to the manufacturer's protocol. 4–6 hours after transfection, cells were trypsinized and replated for cell biological assays. siRNA pool catalog numbers are available in Table S2.

7. Proliferation and Colony Formation Assays

For proliferation assays, cells were plated in 96-well plates at a density of 1000–2500 cells/well and harvested for five days. Cells were fixed and stained with 0.1% crystal violet (VWR, Cat # 97061-850) solution in 20% methanol for 20 min followed by extraction of crystal violet with 10% acetic acid. Absorbance was measured at 600 nm using a plate reader. For colony formation assays, cells were plated in 6-well plates at a density of 1000–2000 cells/well and cultured for 2–3 weeks. Cells were fixed and stained with 0.1% crystal violet (VWR, Cat# 97061-850) solution in 20% methanol for 20 min. Colonies were quantified using ImageJ v1.53c software.

8. Immunoblotting

Protein isolation was performed as previously described [21]. 20μg of total protein were subjected to SDS-PAGE and Western blot, performed as previously described [21]. Primary antibodies used were BRAF (Sigma, , St. Louis, MO, USA, Cat # HPA001328), KRAS (Santa Cruz, Santa Cruz, CA, USA, Cat # sc-30), HA-Tag (Cell Signaling, Danvers, MA, USA, Cat # 3724T), human p53 (Santa Cruz, Cat # sc-126), mouse p53 (BioVision,

Milpitas, CA, USA, Cat # 3036-100), p21 (Abcam, Cambridge, MA, USA, Cat # ab109199), ERK (Cell Signaling, Danvers, MA, USA, Cat # 4695), pERK (Cell Signaling, Danvers, MA, USA, Cat # 9101S), c-Jun (Cell Signaling, Danvers, MA, USA, Cat # 9165S), MAFG (Thermo Fisher Scientific, Waltham, MA, USA, Cat # PA5-90907) and HSP90 (Cell Signaling, Danvers, MA, USA, Cat # 4874).

9. ES Cell Targeting, Mouse Generation, and ESC-GEMM Experiments

ES cell targeting and generation of chimeras was performed a described previously [21]. Embryonic stem cell (ESC)-derived BrafV600E; PtenFL/WT chimeras produced by this approach are topically treated with 4OH-Tamoxifen (4-OHT) to activate melanocyte-specific Tyr-CreERt2, which induces BrafV600E expression and heterozygous Pten deletion (Figure S3D), thereby initiating melanomagenesis. Cre also induces reverse transactivator (rtTA3) expression, enabling melanocyte-specific expression of transgenes upon Doxycycline administration (Figure S3D). Melanoma development was induced in 3–4 weeks old miR-29 sponge and GFP control chimeras having similar ESC contribution using 25mg/mL 4-OH Tamoxifen as described previously [21]. Mice were fed 200 mg/kg Doxycycline (Envigo, Indianapolis, IN, USA, Cat # TD180625) ad libitum. All animal experiments were conducted in accordance with an IACUC protocol (IS00005420) approved by the University of South Florida on 09 May 2018. Experimental mice were euthanized when IACUC-approved clinical endpoints, typically volume of primary tumors, was reached. The derivation of the murine melanoma cell line from an ESC-GEMM chimera was performed as described previously [21].

10. Analysis of miR-29 Target Expression in The Cancer Genome Atlas

We obtained mRNA expression and survival data for KCTD5, MYBL2, SLC31A1, MAFG, RCC2, TUBB2A, SH3BP5L, SMS, and NCKAP5L of 354 skin cancer melanoma tumors from The Cancer Genome Atlas (SKCM-TCGA). To generate a scoring for all nine genes with equal contribution, we normalized the mRNA expression values to the average for each gene, followed by calculating the average of each gene for each patient. For survival analysis, the data was stratified for patients with high or low score according to the median (cutoff: 0.96) and overall survival was estimated according to the Kaplan-Meier method. Groups were compared by the Log Rank test.

11. Statistical Analysis

Statistical analysis was performed using GraphPad Prism8.3 (https://www.graphpad.com/scientific-software/prism/). Survival data were compared by applying the Gehan-Breslow-Wilcoxon test, and all other data were analyzed with the unpaired two-tailed *t*-test or ordinary one-way ANOVA. A *p*-value below 0.05 was considered statistically significant. Experiments were performed in triplicates or quadruplicates and each experiment was repeated at least once. Unless otherwise indicated, one representative experiment is shown. Data represent the mean ± SEM.
